# Social Evolution Selects for Redundancy in Bacterial Quorum Sensing

**DOI:** 10.1371/journal.pbio.1002386

**Published:** 2016-02-29

**Authors:** Eran Even-Tov, Shira Omer Bendori, Julie Valastyan, Xiaobo Ke, Shaul Pollak, Tasneem Bareia, Ishay Ben-Zion, Bonnie L. Bassler, Avigdor Eldar

**Affiliations:** 1 Department of Molecular Microbiology and Biotechnology, Faculty of Life Sciences, Tel-Aviv University, Tel-Aviv, Israel; 2 Department of Molecular Biology, Princeton University, Princeton, New Jersey, United States of America; 3 Howard Hughes Medical Institute, Chevy Chase, Maryland, United States of America; MIT, UNITED STATES

## Abstract

Quorum sensing is a process of chemical communication that bacteria use to monitor cell density and coordinate cooperative behaviors. Quorum sensing relies on extracellular signal molecules and cognate receptor pairs. While a single quorum-sensing system is sufficient to probe cell density, bacteria frequently use multiple quorum-sensing systems to regulate the same cooperative behaviors. The potential benefits of these redundant network structures are not clear. Here, we combine modeling and experimental analyses of the *Bacillus subtilis* and *Vibrio harveyi* quorum-sensing networks to show that accumulation of multiple quorum-sensing systems may be driven by a facultative cheating mechanism. We demonstrate that a strain that has acquired an additional quorum-sensing system can exploit its ancestor that possesses one fewer system, but nonetheless, resume full cooperation with its kin when it is fixed in the population. We identify the molecular network design criteria required for this advantage. Our results suggest that increased complexity in bacterial social signaling circuits can evolve without providing an adaptive advantage in a clonal population.

## Abstract 2

The accumulation of multiple, seemingly redundant, bacterial quorum-sensing systems is promoted by facultative cheating behavior; the strain with multiple systems cheats its single quorum-sensing system ancestor as a minority but returns to cooperation when in the majority.

## Author Summary

Quorum sensing is a mechanism through which bacteria communicate by producing, releasing, and detecting signal molecules encoding information about cell population density. Quorum sensing allows bacteria to synchronize their behaviors and act as collectives. Often, quorum sensing controls cooperative behaviors that benefit the entire community, such as the production and secretion of costly metabolites. Some bacteria release multiple signal molecules which, once detected, funnel information into the same cellular response. Thus, the benefit of using multiple rather than a single signal is mysterious since the signals seem redundant. Here, we combine modeling and experiments to show that the evolutionary accumulation of multiple quorum-sensing systems can be attributed to social exploitation and kin recognition. When in low abundance, a strain that has acquired an additional quorum-sensing system can avoid cooperating and can exploit its ancestor strain, which contains one less quorum-sensing system. The cheater containing the additional system returns to a cooperative behavior when it is abundant. We also identify the molecular mechanisms necessary for the acquisition of an additional signaling system. Our work demonstrates that increased complexity in bacterial social signaling circuits can evolve without providing an adaptive advantage in a clonal population.

## Introduction

Quorum sensing is a mechanism of bacterial cell—cell communication that relies on the production, release, and group-wide detection of extracellular signal molecules called autoinducers. Quorum sensing enables populations of bacteria to coordinate changes in gene expression [1,2].

Bacteria often use quorum sensing to orchestrate the release of public goods (e.g., enzymes or surfactants) whose functions benefit the entire community [2], and to direct other cooperative behaviors such as transitions to more efficient modes of growth [3]. The cooperative nature of quorum sensing is susceptible to exploitation by mutant genotypes that do not contribute to cooperation but benefit from it [2,4–6]. Despite their immediate advantage over the wild-type, exploiting “cheater” genotypes will be eliminated in structured populations due to their negative effect on the average fitness of the community [5,7–11]. In bacteria, population structure can naturally arise in biofilms, where bacteria can grow without significant mixing [10], or during the formation of growth bottlenecks upon invasion into a new environment [11–14].

Many bacterial species employ multiple quorum-sensing systems that impinge on the activity of a shared transcriptional regulator. Each of the quorum-sensing systems encodes a specific receptor and autoinducer production gene with no or limited crosstalk [15]. In several species such as *B*. *subtilis* [16], *V*. *harveyi* [17], and its pathogenic relative, *V*. *cholerae* [18,19], the quorum-sensing systems are arranged in a parallel, seemingly redundant, architecture. That is, all the quorum-sensing autoinducer receptors funnel information into the same signal transduction pathway.

It is unclear what the adaptive benefit is of harboring multiple, rather than a single, quorum-sensing autoinducer—receptor pair when the pairs function in parallel. Here, we combine modeling and experiments in *B*. *subtilis* and *V*. *harveyi* to show that a strain that has accumulated an additional quorum-sensing system reduces its cooperative investment in the presence of its ancestor, but resumes full cooperation in a clonal population. We show that this facultative cheating strategy requires a specific system integration design criterion; the novel receptor must have a dominant repressive effect on the ancestral quorum-sensing response in the absence of the novel autoinducer. We show that, additionally, this particular network design often leads to synergistic activation of the quorum-sensing response by the different autoinducers.

## Results

### Social Selection for an Additional Rap-Phr and against an Additional ComP-ComX Quorum-Sensing System in *B*. *subtilis*

**Fig 1:**
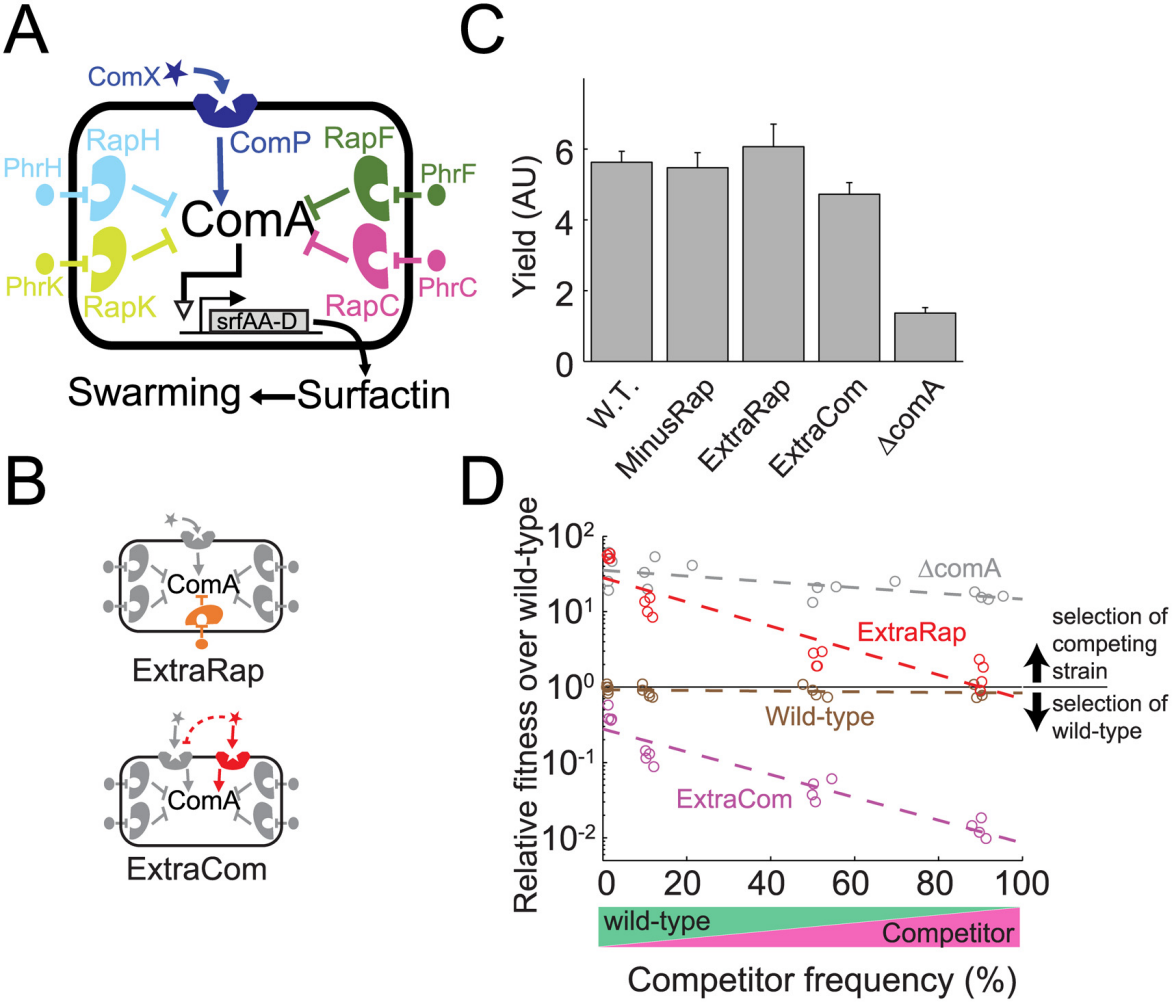
Social selection for accumulation of redundant quorum-sensing systems in *B*. *subtilis*. (A) The *B*. *subtilis* quorum-sensing network controlling ComA is composed of four paralogous Rap receptors, each responding to a specific Phr autoinducer and a single ComP receptor activated by the autoinducer called ComX. Upon binding its cognate ligand, ComP activates ComA. By contrast, Rap receptors are inhibitors of ComA in their unliganged states. (B) Design of two strains carrying additional quorum-sensing systems—ExtraRap harboring the additional orthogonal RapP-PhrP system, and ExtraCom possessing the additional orthogonal ComP-ComX_RO-H-1_ system. The ComP-ComX_RO-H-1_ system inhibits quorum-sensing-controlled gene expression elicited by the endogenous ComP_168_ receptor (red dashed line). (C) Cellular yield, measured by optical density (OD), of the various strains after 48 h of growth on swarm plates. (D) Fitness advantage over the wild-type as a function of the frequency of the competing strain, in a coculture with *ΔcomA* (gray), ExtraRap (red), ExtraCom (magenta), and as a control, wild-type against itself (brown). The cocultures were grown on swarm plates for 48 h. The fitness advantage is the ratio between the relative frequency of the competitor and the wild-type after swarming to that before swarming. Fitness is shown on a logarithmic scale. The data used to produce all figures are provided in S2 Data File.

We hypothesized that social interactions between different genotypes may contribute to the adaptive role of redundant quorum-sensing networks. This hypothesis can be approached by comparing the social behavior of a wild-type species possessing multiple quorum-sensing systems to the behavior of mutant strains harboring varying numbers of quorum-sensing systems. To explore this idea, we first examined the ComA-directed quorum-sensing network of *B*. *subtilis* [16,20–22]. This network is composed of a single ComP-ComX system and multiple paralogous Rap-Phr systems, each encoding its own specific autoinducer. The ComP receptor phosphorylates ComA when ComP is bound to the ComX autoinducer, while Rap receptors repress ComA only when their corresponding Phr ligands are not bound (Fig 1A). All the autoinducers therefore positively control ComA activity, but through different regulatory interactions.

The Com and Rap systems also differ with respect to their population genetics patterns. The ComP-ComX system exhibits significant genetic variability within the population and different alleles form distinct orthogonal signaling pherotypes [20]. These different pherotypes often display partial cross-inhibition, such that an autoinducer from one strain inhibits the response of another strain to its cognate autoinducer [23]. Nonetheless, only a single ComP-ComX system is encoded in each *B*. *subtilis* isolate. By contrast, all *B*. *subtilis* strains encode multiple Rap-Phr systems. The exact number varies between strains, likely due to the association of some Rap-Phr systems with mobile elements [24].

It was previously shown that deletion of any Rap-Phr system has only a small effect on ComA activity, while deletion of the *phr* genes or overexpression of the *rap* genes led to repression of quorum sensing [16,25]. It is therefore unclear why so many quorum-sensing systems regulate ComA, and specifically, why Rap-Phr paralogs proliferate in the genome, while ComP-ComX is unique. To address this question, we constructed strains in which we added to the wild-type strain a novel ComP-ComX system (ExtraCom strain, *comQXP*_*RO-H-1*_^+^ [20]) or we either added (ExtraRap strain, *rapPphrP* ^+^ [26]) or we deleted (MinusRap strain, *ΔrapFphrF*) a Rap-Phr system (Fig 1B, see methods and S1 File for strain construction details). The autoinducing signals produced by the introduced ExtraRap and ExtraCom systems differed from those made by the paralogs present in the parent strain with no cross-activation [20,26] (S1 Fig). We note, however, that the comX_RO-H-1_ autoinducer cross-inhibits the endogenous ComP_168_ receptor [23].

We examined the behavior of these strains under surface-swarming motility conditions, which strictly require the production and release of a ComA-dependent surfactant called surfactin [27,28] (Fig 1C, see methods for details on swarm motility protocol). Unlike the Δ*comA* mutant, the above quorum-sensing variant strains exhibited robust swarming, reaching a similar cell yield as the wild-type after 48 h (Fig 1C, S1A Fig, *p* = 1×10^−5^, F(4,12) = 8.1, *n* = 16; two-way ANOVA for difference between genotypes when including *ΔcomA*, *p* = 0.26, F(3,9) = 8.1, *n* = 12 without *ΔcomA*). Altering the number of quorum-sensing systems therefore does not significantly affect the fitness of the bacteria in clonal populations.

Surfactin may function as a costly public good during swarming, allowing “cheater” strains to exploit the wild-type in coculture. In agreement with this possibility, we found that the *ΔcomA* mutant strain regained its ability to swarm when cocultured with the wild-type, and in so doing, dramatically increased its relative frequency in the population (Fig 1D and S1B Fig; *p* = 10^−4^, two sample *t* test, *n* = 42, see methods for details of the competition experiments and the wild-type competition against itself in Fig 1C that was carried out as a control). When we performed similar coculture experiments between the wild-type and the different quorum-sensing variants, we found that the strain carrying an additional Rap-Phr system was strongly selected for over a strain lacking it. In contrast, the ExtraCom strain was out-competed by the wild-type. Moreover, the fitness advantage of the ExtraRap strain over the wild-type was similar to that of the “cheater” *ΔcomA* mutant at low frequency (*p* = 0.21, t(4,32) = 0.8, linear regression comparison of the intercepts at zero frequency) and approached neutrality as its frequency increased (Fig 1D, *p* = 0.13, t(2,16) = 1.06, linear regression comparison of the intercepts at zero at a frequency of one). Similar results were obtained for wild-type exploitation of the MinusRap strain (S1C Fig).

In contrast to the selection of the ExtraRap strain, the ExtraCom strain remained close to neutral with respect to the wild-type at low frequency, but its competitive disadvantage increased with increasing frequency (Fig 1D, *p* = 10^−8^, t(2,16) = 11, linear regression of slope). Our results are therefore in agreement with the observed population genetics data for the two systems—selection for genomic proliferation of Rap-Phr systems and against proliferation of the ComP-ComX system.

### Mathematical Model Predicts That Facultative Cheating Underlies Social Selection

**Fig 2:**
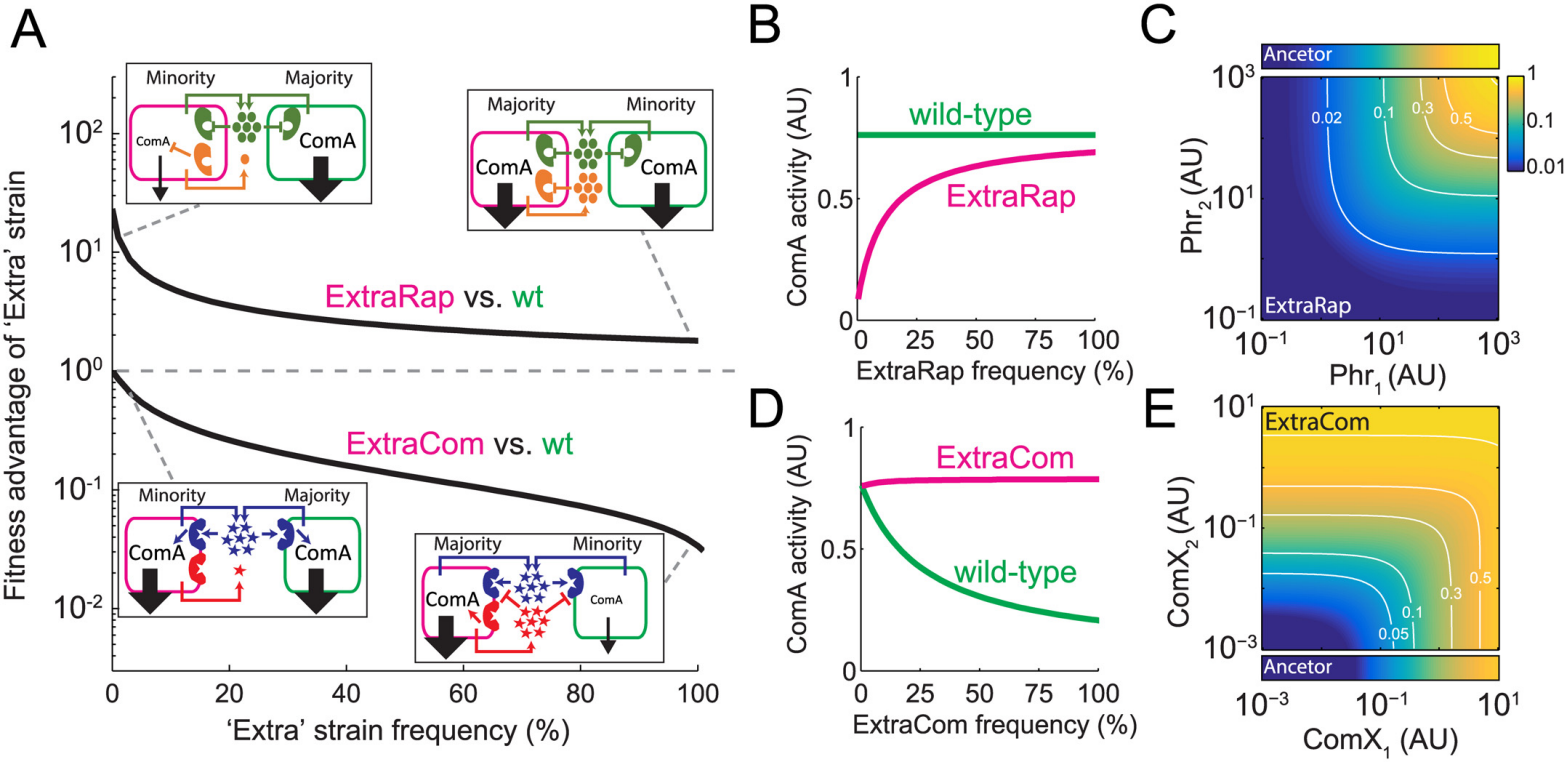
Modeling captures the social interactions in the *B*. *subtilis* ComA network. (A) Modeling results for the fitness advantage of the ExtraRap and ExtraCom strains over the wild-type. Shown in insets are schemes of the social interactions in the extreme cases in which one of the strains is a small minority. Arrow sizes represent the strength of quorum-sensing response of each strain. Compare schemes to Fig 1A and 1B. (B,D) Expected quorum-sensing responses of the ExtraRap (B) and ExtraCom (D) strains and the wild-type, as a function of the frequency of each “Extra” strain when cocultured with the wild-type. (C,E) Two-dimensional regulatory input—output gate of the quorum-sensing response in the ExtraRap (C) and ExtraCom (E) systems. Predicted response to the addition of multiple autoinducers as a function of the levels of the two Phr autoinducers in the ExtraRap strain (C) and the two ComX autoinducers in the ExtraCom strain (E). The narrow bars above the ExtraRap gate (C) and below the ExtraCom gate (E) show the one-dimensional responses of the ancestral strain to the ancestral Phr and ComX autoinducers, respectively. White lines in (C,E) denote equal response curves, and the fold response is provided.

To gain further insight into our results, we mathematically modeled cellular growth and quorum-sensing signaling dynamics during swarming (Fig 2). In the model, we assume a simplified ancestral strain encoding a single ComP-ComX and a single Rap-Phr system. We explored the growth and social dynamics of this ancestor and its corresponding ExtraRap- and ExtraCom-derived strains during swarming (see methods and S1 File for description of the model and its assumptions). Strikingly, the model was able to capture qualitatively the experimental results we obtained above both in clonal and social conditions (Fig 2A and S2 Fig, compare with Fig 1B and 1C). The findings underpin how selection depends on the particular circuit design of the two quorum-sensing systems.

The model also provides simple explanations for the frequency dependence of the ExtraRap system and the difference in selection for and against the ExtraRap and ExtraCom strains, respectively. When a derived “Extra” strain is at low frequency, the concentration of the novel autoinducer it produces is very low compared to those of the ancestral autoinducers, which are produced by all the members in the population (Fig 2A, left insets). In this scenario, the level of quorum-sensing response of the “Extra” strain depends on the activity of the unliganded form of the novel receptor. In the ExtraCom system, the novel ComP receptor is inactive in the absence of its cognate autoinducer. The ancestral network will therefore not be affected by the presence of the novel ComP system, which leads to equal quorum-sensing activation of the ancestral and ExtraCom strains (Fig 2A and 2C). In contrast, in the ExtraRap strain, the autoinducer-free novel Rap receptor represses ComA. Repression is dominant and overpowers activation of ComA by the shared ancestral quorum-sensing system (Fig 2A). Activated ComA levels will therefore be lower in the ExtraRap strain than in the ancestral strain (Fig 2B), leading to selection of the ExtraRap strain due to exploitation of the ancestral strain.

As the frequency of the derived “Extra” strain increases, so does the concentration of its corresponding novel autoinducer (Fig 2A, right insets). In the case of the ExtraRap strain, accumulation of the novel autoinducer leads to partial de-repression of ComA to a level that approaches that of the ancestor (Fig 2B), and this condition occurs as the ExtraRap strain approaches fixation. Therefore, the ExtraRap strain acts as a cheater at low frequency but returns to full cooperation when fixed in the population. In the case of the ExtraCom strain, accumulation of the novel autoinducer leads to a corresponding increase in its quorum-sensing response. In the specific experimental case we examined, the novel ComX system (ComX_RO-H-1_) in the ExtraCom strain cross-inhibits the ancestral ComP_168_ receptor, leading to a strong reduction in ComA activity in the wild-type, ancestral strain (Fig 2C). The ancestral strain therefore acts as a cheater with respect to the ExtraCom strain. Our modeling framework also allows us to explore a theoretical case in which no cross-inhibition occurs. In this situation, the ancestral strain maintains a constant level of ComA activity (S3 Fig). The net selective effect, with or without autoinducer cross-inhibition, is against the ExtraCom system, although selection is stronger when autoinducer cross-inhibition occurs (S3 Fig) [9]. Thus, while autoinducer cross-inhibition naturally exists in the *B*. *subtilis* system we are studying, this feature is not strictly required for selection against accumulation of a novel quorum-sensing system (S3 Fig).

Our model also predicts how the novel autoinducer will act with respect to the ancestral autoinducer in that the model provides us with information about what type of regulatory input—output gate is established. An additional ComP-ComX system leads to formation of an OR-like (additive) regulatory gate for the two ComX autoinducers with respect to their control of ComA activity (Fig 2E). Thus, a single autoinducer is sufficient to elicit a strong quorum-sensing response. In contrast, the repressive activity of an additional Rap system leads to formation of an AND-like (multiplicative) gate between it and the ancestral Phr or ComX system. Thus, the simultaneous presence of both autoinducers is required to elicit a strong response (Fig 2D and S4A Fig).

### Experimental Verification of Facultative Cheating and Autoinducer Input-Output Gate Structures in *B*. *subtilis*

**Fig 3:**
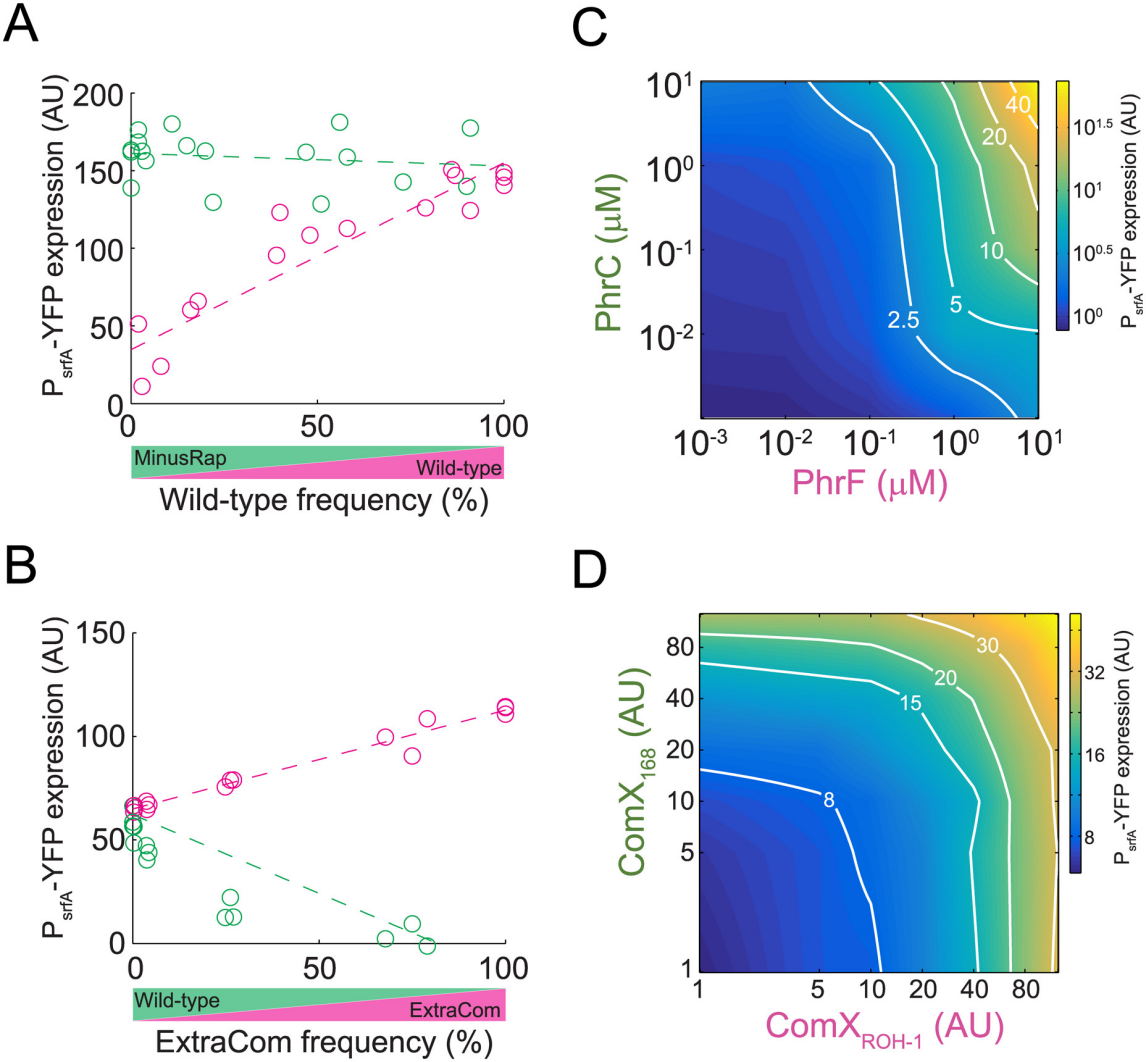
Experimental validation of predicted investment in cooperative behavior in coculture and the resulting regulatory gate of the wild-type. (A,B) Quorum-sensing response of each strain in cocultures of the wild-type (magenta) and the MinusRap (green) (A) and the wild-type (green) and ExtraCom (magenta) (B) strains. The quorum-sensing responses were measured by the fluorescence level of individual cells in which a P_srfA_-YFP reporter was incorporated into the genome of one of the strains (see methods for details). Each circle represents a different experiment. Multiple experiments were repeated on multiple days. (C) The quorum-sensing response gate was measured using the P_srfA_-YFP reporter in a strain deleted for *phrC* and *phrF* and constitutively expressing RapC and RapF. The response was measured at a constant OD for different concentrations of externally added PhrC and PhrF autoinducers. (D) The quorum-sensing response was measured using the P_srfA_-YFP reporter for the ExtraCom strain. Varying levels of conditioned medium prepared from strains harboring only one of the ComP-ComX systems were supplied to a low-cell density population of ExtraCom cells. Shown in both (C,D) is a heat map of a continuous interpolation of the data (original data are shown in S6A and S6C Fig and S1 Data). The equal-response lines are marked in white and indicated by their fold-response values.

Our model predicts that the different architectures of the two quorum-sensing systems lead to differential investment in cooperative behavior by the ancestral and derived strains as well as to distinct regulatory input—output gate structures. These features result in the observed patterns of selection. To address these predictions experimentally, we introduced a YFP transcriptional reporter for ComA activity (P_*srfA*_-YFP) into the wild-type, the ExtraCom, and the MinusRap strains. We cocultured each reporter-containing strain with a reporter-free counterpart and measured gene expression as a function of frequency (Fig 3A and 3B and S5A Fig). To minimize the effect of changes in frequency and spatial distribution, we performed these assays in minimal medium using a surfactin production-deficient mutant of the *sfp* gene (*sfp* ^−^ [27]), which has reduced quorum-sensing-associated cost. Similar results were obtained when gene expression was measured during swarming (S5B Fig). The absence of surfactin in the minimal growth medium did not significantly affect expression of the P_*srfA*_-YFP reporter construct in the cocultured strains (S5B Fig). We found that, when the MinusRap and wild-type strains are cocultured, the MinusRap strain maintained a constant ComA activity, irrespective of its frequency in the population (F(1,16) = 0.41, *p* = 0.53, *n* = 18, linear regression of the slope). In contrast, at low frequency, the wild-type exhibited low level ComA activity, which increased with increasing frequency of the wild-type (F(1,15) = 96, *n* = 17, *p* < 10^−7^, linear regression of the slope). At high frequencies, wild-type ComA activity approached the activity level of the MinusRap strain (Fig 3A, *t* test, *p* = 0.43 for interception of best-fitted lines at a frequency of 1).

When the ExtraCom strain was cocultured with the wild-type, ComA activity was the same in both strains when the frequency of the ExtraCom strain was low (*t* test for linear regression of lines, *p* = 0.26 for interception at frequency of zero). ComA activity in the ExtraCom strain increased with increasing frequency (Fig 3B, *p* = 10^−11^ F(1,14) = 405, for a zero slope). In accordance with the expected effects of crossinhibition (Fig 2 and S3 Fig), the ComA activity of the wild-type strain decreased dramatically with increasing frequency of the ExtraCom strain (Fig 3B, *p* = 10^−6^ F(1,15) = 54, for a zero slope).

We next measured the resulting regulatory gate structure of the response of ComA to addition of multiple autoinducers. We constructed a strain constitutively expressing the *rapF* and *rapC* receptor genes but not their respective *phrF* and *phrC* autoinducer-production genes. We found that the ComA response was significant only if both the PhrF and PhrC autoinducers were present, showing an AND-like gate structure (Fig 3C, S6A and S6B Fig). Likewise, an AND-like response occurred for PhrF and ComX regulation of ComA (S5 Fig). In contrast, regulation of ComA by the two ComX autoinducers in the ExtraCom strain was additive as expected for an OR-like response (Fig 3D, S6C, S6D and S6E Fig, methods).

### A General Design Criterion for Accumulation of Quorum-Sensing Systems

The experimental results support the role of social interactions in selection for accumulation of Rap-Phr systems coupled with selection against accumulation of ComP-ComX systems in *B*. *subtilis*. In order to generalize these results, we formulated a generic model of selection with respect to quorum-sensing-dependent public goods (S1 File). This model suggests that two design criteria are necessary and sufficient for the invasion of a strain carrying an additional quorum-sensing system into a population lacking it: 1) Dominant repression: The ligand-free novel receptor should act negatively to overpower the quorum-sensing response of the ancestral system, and 2) Facultative operation: The addition of the novel autoinducer should restore the quorum-sensing response to levels similar to that of the ancestor. The combination of these two features allows the invading strain to perform facultative cheating—cheat the ancestor in coculture (criterion #1) but resume cooperation when it is fixed in the population (criterion #2) [9,29].

In the S1 File, we show that if repression by the novel quorum-sensing system is strong, the two autoinducers will regulate the response in an AND-like manner. We further demonstrate that formation of an AND-like gate is sufficient but not mandatory to select for acquisition of a novel quorum-sensing system. Likewise, an OR-like gate between autoinducers is sufficient but not required to select against the acquisition of a novel quorum-sensing system. The AND-like and OR-like gate structures provide an intuitive, albeit simplified, explanation for selection (AND) or counterselection (OR) of an evolved strain. If both autoinducers are necessary to activate the quorum-sensing response in the evolved strain (AND gate), while the ancestral strain produces and responds to only one of the autoinducers, then the evolved strain will cease to cooperate when present as a small minority together with its ancestor. In contrast, if either autoinducer is sufficient, then the evolved strain will continue to cooperate even when it is present as a minority.

### Accumulation of Quorum-Sensing Systems Leads to Facultative Cooperative Investment in *V*. *harveyi*

**Fig 4:**
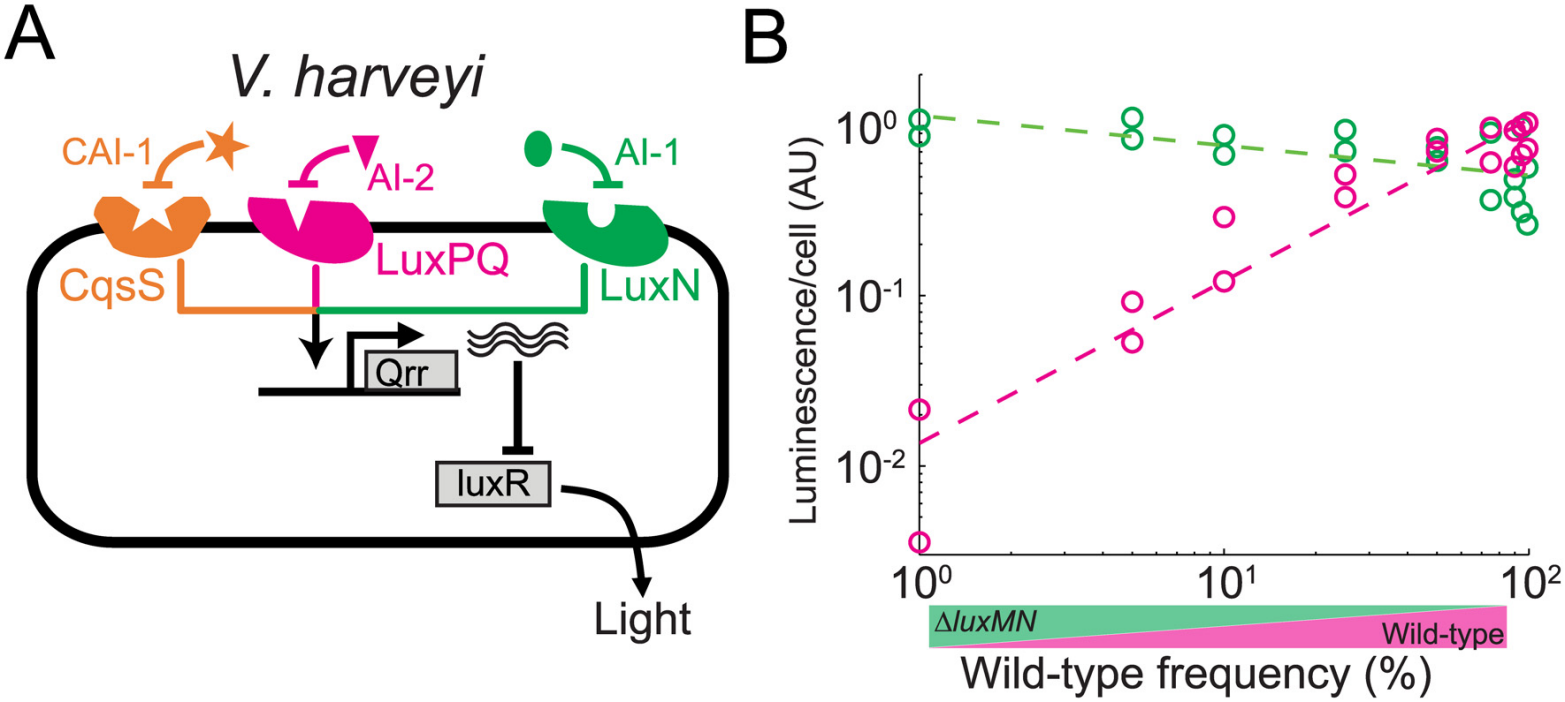
Facultative quorum-sensing response in *V*. *harveyi*. (A) Schematic structure of the architecture of the *V*. *harveyi* quorum-sensing network. (B) Quorum-sensing response in *V*. *harveyi* was measured using the natural quorum-sensing-dependent bioluminescence output. Shown is the bioluminescence per cell as a function of wild-type frequency, for the wild-type (magenta) and the *luxMN* mutant (green) during coculture of the two strains. Bioluminescence of each strain was measured by introducing a null mutation into the *lux* operon of the cocultured partner strain.

We next examined whether our results also apply to another well-studied model organism in which multiple quorum-sensing systems exist and control a common output. The bioluminescent marine bacterium *V*. *harveyi* [15] has a quorum-sensing network composed of three parallel systems that regulate expression of the quorum-sensing master transcription factor LuxR, which controls multiple traits including bioluminescence emission (Fig 4A). A similar architecture composed of four quorum-sensing systems exists in the related pathogen, *V*. *cholerae* [19]. While the deletion of any of the receptors does not affect the quorum-sensing response [18], deletion of any of the autoinducer synthase genes represses LuxR-activated genes, demonstrating the dominant repressive effect of each ligand-free receptor [17]. In addition, two of the autoinducers have been shown to act multiplicatively in their regulation of LuxR [30] and to synergistically control bioluminescence [31]. We used the abundant quantitative data on this organism to construct a model of the expected social behavior of the wild-type and an ancestral-like strain deleted for any one of the quorum-sensing systems (S1 File, S7A Fig). The model predicts that the wild-type will reduce its cooperative investment in the presence of such an ancestral strain, which will lead to facultative cheating under appropriate conditions.

To verify the model experimentally, we constructed a putative ancestral strain deleted for the *luxMN* autoinducer-receptor system. We introduced a null mutation into the *lux* (luciferase) operon in the wild type and derived ancestral strains, and by mixing Lux^+^ and Lux^−^ pairs (WT/Lux^+^ mixed with *luxMN*/Lux^−^ and WT/Lux^−^ mixed with *luxMN*/Lux^+^), we could measure the level of quorum-sensing response per cell of the Lux^+^ strain in each coculture (Fig 4B and S7B Fig, methods). As expected from the model, we found that light production by the *luxMN* mutant strain remained almost constant irrespective of its frequency (the small decrease is most likely due to the effect of the *lux* locus, S7B Fig). The wild-type showed a near 100-fold reduction in bioluminescence output compared to the *luxMN* mutant at low frequency (*p* < 10^−13^, T(32) = 12.7, *t* test on linear regression for intersect at zero frequency), while approaching the same level of light production as the *luxMN* mutant at high frequency. Our rationale therefore also applies to the parallel quorum-sensing network of *V*. *harveyi*.

## Discussion

In this work, we propose that bacteria possessing multiple quorum-sensing networks that control the identical response, which are commonly found in nature, are selected through a facultative cheating process. Facultative cheating has been described in the past as a strategy by which microorganisms exploit nonkin but return to cooperation in the presence of kin [32]. Such behavior has been described in fruiting body-forming amoeba and bacteria [29,33], but the underlying molecular processes that lead to it are unknown [34]; however, links to cell—cell signaling and facultative cheating have been suggested [35–37].

We predict that accumulation of multiple quorum-sensing systems requires a specific set of network design criteria, the functioning of which we explored in two diverse but well studied organisms. Specifically, the introduced novel receptor must repress the quorum-sensing response in the absence of the novel autoinducer, as occurs in the *B*. *subtilis* Rap-Phr and *V*. *harveyi* Lux quorum-sensing systems. In contrast to these systems that depend on repression, other quorum-sensing systems exist that act positively, in that the receptor functions as an activator upon autoinducer binding. Our model and experimental results explain why accumulation of parallel positively acting systems is selected against. Indeed, we do not know of any bacterium that possesses multiple activation-based quorum-sensing systems that function in parallel. Rather, activation-based systems are commonly organized in a hierarchy, in which one quorum-sensing system regulates the expression of a second system. A hierarchical network design is not fully redundant because the two quorum-sensing systems can control different genes. Further work will be required to define the benefits and possible evolutionary routes giving rise to quorum-sensing systems that function positively and are arranged as hierarchies.

Beyond facultative cheating, other possible adaptive functions for possessing multiple quorum-sensing systems have been suggested. These include gains in information acquired about cell density, information about the frequency of phenotypes in the vicinal population, and access to information about physical flow conditions [38–41]. Our social selection model does not contradict those alternatives and may promote them by driving the initial fixation of the redundant network design, which can, subsequently, be further modified for other adaptive advantages.

Several processes may limit the accumulation of quorum-sensing systems. First, each system contributes a signaling cost [5,42]. Second, the facultative return to cooperation may not be complete, leading to reduced benefit during exploitation in structured populations. Third, social selection of facultative characters is weak and can lead to variable mutation selection balance [34]. Finally, rareness of available systems and the need to integrate them appropriately into the existing network may limit the rate of accumulation. Further work will be required to define the importance of each of these mechanisms.

Exploitation can also occur between species; not only between variants within species. For example, cooperative secretion of antibiotic degrading enzymes has been shown to lead to coexistence of secreting and nonsecreting genotypes, at both the species and interspecific levels [43,44]. Accumulation of additional quorum-sensing systems could also be used to exploit species that produce fewer signals. This ecological factor may contribute to the continuous selection for maintenance of multiple systems. More generally, our results point to the roles facultative cheating and kin recognition may have in the ecology of complex microbial communities.

## Materials and Methods

### Growth Media and Conditions

Routine growth was performed in Luria—Bertani (LB) broth: 1% tryptone (Difco), 0.5% yeast extract (Difco), 0.5% NaCl. Experiments with *B*. *subtilis* were done using Spizizen minimal medium (SMM): 2 g L^−1^ (NH_4_)_2_SO_4_, 14 g L^−1^K_2_HPO_4_, 6 g L^−1^KH_2_PO_4_, 1 g L^−1^disodium citrate, 0.2 g L^−1^MgSO_4_∙7H_2_O. This was supplemented with trace elements (125 mg L^−1^MgCl_2_∙6H_2_O, 5.5 mg L^−1^CaCl_2_, 13.5 mg L^−1^FeCl_2_∙6H_2_O, 1 mg L^−1^MnCl_2_∙4H_2_O, 1.7 mg L^−1^ZnCl_2_, 0.43 mg L^−1^CuCl_2_∙4H_2_O, 0.6 mg L^−1^CoCl_2_∙6H_2_O, 0.6 mg L^−1^ Na_2_MoO_4_∙2H_2_O). Unless otherwise noted, 0.5% glucose was used as carbon source. Petri dishes for routine procedures were solidified using 1.5%agar (Difco).

Antibiotic concentrations: Macrolides-lincosamides-streptogramin B (MLS; 1 μg ml^−1^ erythromycin, 25 μg ml^−1^ lincomycin); Spectinomycin (Sp, 100 μg ml^−1^); Tetracycline (Tet, 10 μg ml^−1^ for *B*. *subtilis*); Kanamycin (Km, 5 μg ml^−1^); Chloramphenicol (Cm, 15 μg ml^−1^); Ampicillin (Amp, 100 μg ml^−1^); Carbenicillin (Carb, 300 μg ml^−1^). Isopropyl β-D-thiogalactopyranoside (IPTG, Sigma) was added to the medium at the indicated concentration when appropriate.

Premeasurement Bacillus growth protocol: Prior to all measurements, an overnight colony from an LB agar plate was inoculated in 1 mL SMM liquid medium and grown for 7 h until an OD_600_ of 0.1–0.3 was reached. The cultures were diluted by a factor of 10^6^ and grown overnight at 37°C. Overnight cultures were centrifuged, resuspended in PBS, and diluted to an OD_600_ of 0.01. We find that this long incubation in minimal medium both reduced the effects of quorum sensing prior to growth and reduced the arbitrary difference in growth between two cocultured wild-type colonies.

For coculture experiments, cells of different strains were mixed in appropriate ratios after overnight growth in SMM, based on relative optical density (OD). The exact ratios were subsequently measured using flow cytometry.

### Peptide Synthesis

Synthetic pentapeptides PhrF (NH2 QRGMI COOH) and PhrC (NH2 ERGMT COOH) were purchased from GL Biochem (Shanghai, China) at >98% purity. 10 mM aliquots were prepared by resuspension of the lyophilized peptides in H_2_O and stored at −20°C.

### Flow Cytometry

Samples were analyzed on a Gallios flow cytometer (Beckman-Coulter), equipped with four lasers (405 nm, 488 nm colinear with 561 nm, 638 nm). The emission filters used were: BFP– 450/50, YFP/GFP– 525/40, mCherry– 620/30. Events were discriminated using the forward-scatter parameter. For each run, discrimination enabled a single, well-defined population to appear in the forward-scatter (FS) by side-scatter plot. Gating on the fluorescent populations and inspection of the nondiscriminated forward by side-scatter plot indicated that over 99.9% of the fluorescent cells are present in the discriminated population. In all analyzed samples, only single cells were considered by gating on correlated time-of-flight and FS events. Gating of the different fluorescent populations was performed by inspection of the log-log FLx by FLy plots (where *x* & *y* represent the appropriate filter number for each fluorescent marker), where two distinct populations were clearly visible, resulting in type-I and type-II errors of less than 0.05%. For each run, at least 100,000 cells were analyzed and the total events analyzed such that the minority population was never below 1,000 events.

### Swarming Experiments

Cells were grown as described in the premeasurement growth protocol. Five microliters of diluted cultures were placed at the centers of 0.7% agar plates containing 25 mL of SMM medium supplemented with trace elements and 0.05% glucose. The plates were prepared 1 h prior to inoculation, allowed to solidify in room temperature, and dried for 5 min in a laminar flow chamber. The plates were incubated at 30°C for up to 72 h. The swarms were collected after suspension in 5 ml of PBS, the OD was measured, and the final ratios or the gene expression was determined using flow cytometry as described above. For spatial analysis of swarming, samples were taken from the centers of the plates, in addition to several samples 1 cm and 2 cm from the center. We find that a glucose concentration of 0.05% compared to 0.5% reduced the residual swarming of the *comA* mutant, increasing the difference in growth between the mutant and the wild-type, likely because residual production of surfactin by the mutant colony was reduced.

### *B*. *subtilis* Gene Expression Experiments

In all experiments, YFP level was determined from the median level of the unimodal distribution of YFP expressing cells using flow cytometry. YFP level was normalized by the autofluorescence of the wild-type. For coculture experiments (Fig 3A and 3B), samples were taken at several time points, and the OD_600_ and YFP levels were measured by flow cytometry. The expected YFP level at OD_600_ = 1 was calculated by interpolation.

#### Regulatory gates

Three separate experiments were performed to measure the P_*srfA*_-YFP expression levels as a function of dual combinations of different autoinducers (Fig 3C and 3D and S4 Fig). In each of the experiments, the relevant strain was grown to a set OD, when different concentrations of the relevant autoinducers were added to different aliquots and incubated for 90 min, after which YFP was measured using flow cytometry. For the PhrF-PhrC gate (Fig 3C), cells of strain AES2636 were grown to OD of 0.6 when different concentrations of the two synthetic peptides were added to different aliquots. For the ComX_ROH-1_-ComX_168_ gate (Fig 3D), cells of strain AES2278 were grown to OD ~0.001. Different 250 μl aliquots were then complemented with 250 μl of a composite conditioned media. Composite conditioned media was composed of varying fractions of conditioned media prepared from three strains: AES2202 (producing ComX_RO-H-1_), AES2277 (producing ComX_168_) and AES2135 (not producing ComX), which were grown to OD_600_ of 1.0 prior to preparation of the conditioned media. For the PhrF-ComX gate, cells of strain AES2672 were grown to an OD_600_ of ~0.001. Aliquots were then supplemented with varying concentrations of PhrF and 250 μl aliquots of a composite conditioned media mixture of conditioned media from strains AES2277 and AES2135. The PhrF-ComX_168_ and ComX_ROH-1_-ComX_168_ gates were measured at low OD_600_ to prevent accumulation of ComX autoinducers produced by the reporter strains (which are not deleted for the *comX* genes).

### *V*. *harveyi* Coculture Assays

*V*. *harveyi* strains were grown at 30°C in Luria-marine (LM) medium with aeration. Following overnight growth, samples were diluted to OD_600_ = 0.005 with varying ratios of dark and bright strains. Following 6.5 h of growth, bioluminescence was measured on a Tri-Carb 2810 TR (Perkin Elmer) scintillation counter. Dilutions of the cultures were made and plated on LM agar plates. Plates were incubated at 30°C overnight to allow colony formation. Images of the plates were taken using an ImageQuant LAS system that detects both bioluminescence and total colony forming units (CFUs). Colonies were counted using the ImageQuant TL and ImageJ programs. Values shown are calculated as (total bioluminescence)/(# bioluminescent CFUs). The values were normalized to the bioluminescence per cell of the bright strain.

### Strain Construction

All strains are detailed in S1 Table, while respective primers are provided in S2 Table.

### *B. subtilis*

All of the mutations and constructs were transferred to PY79 by transformation [45]. Integration of *amyE* integration plasmids into the *zjd89*::*amyEΩ Cm Km* [46] was done as previously described [26].

Deletion of *rapF-phrF*, *rapC-phrC*, *comA*, and *comQXP* from the PY79 chromosome and their replacement with the MLS resistance cassette was performed through the long flanking homology PCR method [47] using the primers rapF-P1-P4, rapC-P1-P4, comA-P1-P4, and comQXP-P1-P4, respectively (S2 Table). The *rapFphrF*::Cm deletion was generated using the antibiotic switching vector ece76. *rapFphrF*::Cm was next used as a template to generate *rapFphrF*::Tet using the antibiotic switching vector ece75 (S1 Table).

To generate inducible *zjd89*::*(P*_*hyperspank*_ *-rapF)* and *amyE*::*(P*_*hyperspank*_ *-rapC)* constructs, a PCR product containing the relevant open reading frame was amplified using the primer pairs hsRapF-F/hsRapF-R andhsRapC-F/hsRapC-R. The PCR products were digested with the appropriate enzymes (S2 Table) and ligated downstream of the hyperspank promoter of the pDR111 vector containing Spec resistance [48].

Construction of *sacA*::(comQXP_RO-H-1_ Cm) was performed by PCR amplification of *comQXP* from strain *B*. *mojavensis* RO-H-1 using the comQXP-ROH1-F and comQXP-ROH1-R primer pair. The PCR product was digested with restriction enzymes BamHI and EcoRI and ligated to the ece174 plasmid (S1 Table). The resulting vector was integrated into the *sacA* site on the chromosome using Cm resistance for selection.

Construction of *sacA*::(*P*_*srf*_-*3xyfp* Cm) was performed by PCR amplification of P_*srf*_-3xyfp using AEC945 as a template and the Psrf-sacA-F/Psrf-sacA-R primer pair. The PCR fragment was digested with the appropriate enzymes (S2 Table) and ligated to the ece174 plasmid. The resulting vector was integrated into the *sacA* site on the chromosome using Cm resistance for selection.

The *swrA* ^+^ mutation allele is a spontaneous revertant that was selected by plating *swrA* ^−^ *sfp* ^+^ cells on 0.7% LB agar plates and selecting motile variants, as was done previously [28]. The reconstituted *swrA* ^+^ allele was verified by sequencing.

The *sfp*+ allele was amplified from the undomesticated strain *B*. *subtilis* NCBI3610 and fused to a spectinomycin resistance cassette by PCR using primers sfp-P1-P4 (S2 Table).

The constitutive fluorescent construct P_43_-*yfp* was synthesized by Genewiz, and sub-cloned into ece137 using BamHI and EcoRI restriction enzymes.

### *V. harveyi*

Plasmid pBB1131 (pLAFR2/*luxCDABE*::Tn*5*) was conjugated into strains BB120 (WT) and HLS252 (Δ*luxMN*). The *luxCDABE*::Tn*5* region was transferred to the endogenous *luxCDABE* locus on the chromosome to generate the dark strains. In pBB1131 the Tn*5* is located in the *luxA* gene.

### Modeling

Modeling of social interactions, signaling, and growth of the different organisms was done using regular differential equations, which describe the kinetic interactions between the molecular components of the quorum-sensing signal transduction process, cell growth kinetics and its dependence on nutrient availability, and public goods production. The equations were either analytically treated or solved numerically using Matlab (Mathworks). The specific equations used and a discussion of their relevance to the known biological data are provided in S1 File.

## Supporting Information

S1 Fig Growth and selection during *B*. *subtilis* swarming. (A) Shown are cell yields of the indicated genotypes after 24 (light gray) and 48 (dark gray) h. 48 h results are reproduced from Fig 1C of the main manuscript. The 24 h differences are not statistically significant given the sample size. (B) Spatial structure of invasion. Cocultures of the indicated strains at the indicated frequencies were inoculated onto swarm plates. 48 h after inoculation, the frequencies of the strains were measured at different positions on the plates—center (one sample per plate) and at a distance of 1 or 2 cm from the center (four samples per plate). Each experiment was repeated twice. Shown are the averages and standard deviations of the apparent fitnesses at the different positions and for the different strain compositions. Apparent fitness is defined as the relative frequency at the position of sampling divided by the initial relative frequency of the two strains. The lower apparent fitness at a distance of 2 cm agrees well with the known *B*. *subtilis* swarming mechanism—cells initially swarm as a thin monolayer, and then they grow. The reduced apparent fitness stems from the lower number of doublings. (C) Shown are the relative fitness values of wild-type over the MinusRap (green) and ExtraRap over the wild-type (red, reproduced from Fig 1D). Fitness is shown as the function of the invading strain's initial frequency. (D) Specificity of Rap-Phr systems. Shown are the YFP levels of the P_*srfA*_-YFP construct for strains overexpressing RapF, RapC, or RapP 3 h after the exogenous addition of no autoinducer (blue) or 10 μM of the PhrC (cyan), PhrF (orange), or PhrP (brown) autoinducers. Each Rap responds specifically to its cognate autoinducer. RapC and RapF overexpression constructs were introduced into a *ΔrapF-phrF*, *ΔrapC-phrC* deletion background, while RapP was introduced into the wild-type background.
(EPS)

S2 Fig Characterization of the mathematical model of the *B*. *subtilis* ComA quorum-sensing network. (A) Final cell yields of the different strains at time point T = 800 (compare with B). (B) Cell number (yield) as a function of time during clonal growth of the four strains (see legend). The three derived variants terminate growth once they expand into the maximal area, while the Δ*comA* mutant continues to grow. (C) Absolute frequency as a function of time during co-culture of the ExtraRap (red), ExtraCom (purple) and Δ*comA* (gray) strains with the wild-type. Initial frequency of the three strains is 1%. (D) Levels of active ComA as a function of time for each of the strains in a coculture of the ExtraRap (red) and wild-type (brown) strains with an initial frequency of 1% ExtraRap. See S1 File for further details.
(EPS)

S3 Fig Modeling the effect of cross-inhibition between ComX autoinducers. The ComX_ROH-1_autoinducer was previously reported to suppress the response of ComP_168_ to the ComX_168_ autoinducer [23]. We model this phenomenon by assuming strong competitive inhibition between the autoinducers (see S1 File). (A) Shown are the model’s predictions for the fitness advantage of the ExtraCom strain over the wild-type strain in coculture. Fitness advantage is defined in the caption to Fig 1 of the main manuscript. Two cases are considered, with crossinhibition (continuous line, also as shown in Fig 2 of the main manuscript) and without crossinhibition (dashed line). (B,D) Gene expression of the wild-type strain and the ExtraCom strain as a function of frequency for the model lacking cross-inhibition (B) and including crossinhibition (D, replicated from Fig 2 of the main manuscript). (C,E) Response of the ExtraCom strain to addition of the two types of ComX autoinducers, with (E) and without (C) cross-inhibition. See S1 File for further details.
(EPS)

S4 Fig Rap-Com regulatory gate—model and experiment. (A) Results of the *B*. *subtilis* mathematical model for the level of active ComA in the wild-type when varying levels of ComX and Phr autoinducers are added. The response is purely multiplicative to a good approximation—*f*(*Phr*, *ComX)*~*h*(*Phr* × *ComX*). See further mathematical treatment and discussion in S1 File. (B) Raw data for the experiment in which ComA responses are measured following addition of varying concentrations of the PhrF and ComX autoinducers. *ΔphrF* cells that constitutively express *rapF* were grown to low cell density. PhrF autoinducer peptide was directly added in varying amounts. ComX autoinducer was added at varying amounts by supplying a constant volume of conditioned medium made from particular fractions of wild-type- and *ΔcomQXP*-conditioned media. The response was measured using the P_*srfA*_-YFP reporter. Note the logarithmic scale of the *x*, *y* axes. (C) Interpolation of the raw data is presented as a two-dimensional heat map. White lines indicate equal response curves at the designated response levels. The curves fit nicely to a straight line of angle ~45°, as expected from a multiplicative function. (D) Synergy of the ComX-PhrF regulatory gate. For any given level of the autoinducers, the similarity to an AND gate can be assesed by the level of synergy of the response, which is defined as the value of the response for that specific combination of autoinducers, divided by the sum of the two responses when only one of the autoinducers is supplied at the identical concentration. Shown are the synergy levels as a function of the strength of the response for the data presented in panel B (red) and for a repeat of the experiment in which the raw data are not shown (purple). The dashed line at a synergy value of 1 indicates the expected level from an additive model, *f*(*s*_1_, *s*_2_) = *f*(*s*_1_, 0) + *f*(0, *s*_2_). See further discussion on synergy and its relation to the establishment of particular regulatory gates in S1 File. We note that in some of the experiments, we did not measure the response for zero concentration of some of the autoinducers. In such cases, we used the response with minimal level of autoinducer as an estimation for the response with zero autoinducer. The calculated synergy levels in these cases are therefore underestimates of the actual synergy.
(EPS)

S5 Fig Measurement of gene expression in coculture in minimal medium and during swarming. (A) Examples of gene expression trajectories as a function of cell density for the wild-type (magenta) and MinusRap (green) with the indicated initial frequencies of the specific strain in the coculture. The measured strain encodes the P_*srfA*_-YFP reporter, while the other strain in coculture encodes a constitutive mCherry reporter. The graphs were interpolated to identify the predicted expression level at an OD of 1. This level of expression was used in Fig 3 of the main manuscript. (B) Left: The swarming proficient MinusRap and wild-type strains were mixed at the indicated levels as in the minimal medium experiment. Gene expression was measured after 48 h from the entire plate. The wild-type expression when the strain is at low frequency is lower than the MinusRap expression when it is the majority strain by a factor of ~8 (*p* = 5 × 10^−5^), while the two strains exhibit the same level of expression when the wild-type strain is present at a majority (*p* = 0.26). Right: The experiment shown in Fig 3 of the manuscript was repeated when surfactin was added at concentration of 5 μg/ml to the minimal medium. Shown are results for coculture with 1% of the wild-type or 99% of the wild-type. Asterisks mark statistically significant differences.
(EPS)

S6 Fig Analysis of the PhrF-PhrC and ComX_168_-ComX_ROH-1_ experimentally defined regulatory gates. (A,C) Raw data on which the interpolations shown in Fig 3C and 3D of the main manuscript are based. (A) PhrF-PhrC regulatory gate, (C) ComX_168_-ComX_ROH-1_ regulatory gate. (B,D) Synergy levels of the two gates presented in (A,C). Synergy is defined and discussed in the caption of S3 Fig. Purple circles in (B) show a repeat of the experiment. (E) Interpolation of the ComX_168_-ComX_ROH-1_ data shown in (C) onto a linear scale. White lines represent equal responses at the indicated values. These lines fit well to a linear model, indicating an additive response function for the two autoinducers, *f*(ComX_168_, ComX_ROH−1_) = *h*(*α*ComX_168_+βComX_ROH−1_). See further discussion on additive responses in S1 File.
(EPS)

S7 Fig Model prediction of for the *V*. *harveyi* LuxR regulatory gate. Predicted LuxR levels (in arbitrary units) for the addition of different concentrations of the AI-2 and AI-1 autoinducers (in nM) for a strain expressing both receptors (main graph), or for mutants lacking the LuxPQS system (top) or the LuxMN system (right). This analysis is based on refs. [49,50], as explained in S1 File. Compare the main panel here to the corresponding experimental panel in Fig 4B of ref [30]. See further details on the analysis in S1 File. (B) Shown are the bioluminescence levels of cocultures of bioluminescent (*lux* ^+^) and mutated (*lux* ^−^) strains, as a function of the frequency of the *lux* ^+^ strain. This includes the cocultures of the wild-type and the *ΔluxMN* strains which are shown in Fig 4E of the main manuscript (magenta and green). Note that the coculture in which the *ΔluxMN* is *lux* ^*+*^ (green), is an increasing function, as the *x*-axis represents the *ΔluxMN* frequency and not the wild-type frequency. Also shown are two controls in which the wild-type (blue) and the *ΔluxMN* (orange) *lux* ^+^ strains where cocultured with a *lux* ^−^ variant of the same genetic background. The bioluminescence patterns of the two controls and the coculture of *ΔluxMN*, *lux* ^*+*^ and wild-type, *lux* ^−^ are indistinguishable (*p* = 0.24, F(54,2) = 1.43, for three lines to be the same assuming parallel lines, using ANCOVA analysis on genotype by frequency). This result indicates that the small change in the frequency of the strains is due to the effect of the *lux* locus and not of the *luxMN* locus.
(EPS)

S1 File Supplementary text. (DOCX)

S2 File Excel spreadsheet containing the experimental data shown in the manuscript and supplementary figures. (XLSX)

S1 Table Strain list. (DOCX)

S2 Table Primer list. (DOCX)

## Abbreviations

CFU: colony forming unit
FS: forward-scatter
LM: Luria-marine
OD: optical density
