# Supplementary material for: Social Evolution Selects for Redundancy in Bacterial Quorum Sensing
Source: PLoS Biol. 2016 Feb 29;14(2):e1002386. doi: 10.1371/journal.pbio.1002386 (PMC4771773; doi:10.1371/journal.pbio.1002386)
Supplement: S1 File — (DOCX) [file pbio.1002386.s008.docx]

Supplementary text for

"Social evolution selects for redundancy in bacterial quorum sensing"

Eran Even-Tov^1+^, Shira Omer^1+^, Julie Valastyan^2^, Xiaobo Ke^2^, Shaul Pollak^1^, Tasneem Bareia^1^, Ishay Ben-Zion^1^, Bonnie L. Bassler^2,3^ and Avigdor Eldar^1*^

Table of Contents

**Mathematical modeling: *Bacillus subtilis*** **2**

**Mathematical modeling: *Vibryo harveyi*** **10**

**Mathematical modeling: Generalization** **13**

**Supplementary references17**

Below, we formulate specific models for the social dynamics and quorum-sensing network response of wild-type and engineered strains of *Bacillus subtilis* and *Vibrio harveyi*. We demonstrate that mathematical modeling predicts the invasion and expression patterns we observed in our experiments. Thus, the modeling allows us to gain a mechanistic understanding of the design principles that dictate the social interactions between quorum-sensing variants and their resulting evolutionary fates. These specific mathematical models serve as the basis for the simulation data shown in Fig. 2 of the main manuscript and S2,S3,S4 and S7 Fig.

We also present a generalization of our analysis to a specific model which allows us to validate the general design criterion that promotes invasion of a strain with a novel quorum-sensing system into a population: the novel receptor should repress the quorum-response in the absence of its cognate autoinducer. Finally, we show that this design criterion, coupled with the inclusion of several biologically realistic assumptions, will lead to synergistic interaction between the autoinducers in the wild-type strain with regard to regulation of the quorum-sensing response.

# Mathematical modeling: *Bacillus subtilis*

As explained in the main text, *B. subtilis* has two types of quorum-sensing systems - the ComPX system and the Rap-Phr system. Here, we describe the details of the molecular mechanisms of each system, and we define the simplifying assumptions that will be used in the modeling. We subsequently write the model equations and analyze them both numerically and analytically.

## General information about *B. subtilis* quorum sensing

**ComPX quorum-sensing system**. The ComPX system involves three genes organized in a single operon, *comQ*, *comX*, and *comP*. ComP, the receptor of the system, is a 10-transmembrane-spanning protein whose autoinducer binding domain is most likely located in the first outer-membrane loop [[1](#_ENREF_1)]. ComP functions as a receptor histidine kinase, in which binding of the ComX autoinducer leads to autophosphorylation of the ComP histidine kinase domain and phospho-transfer to ComA. ComA is the only known target of ComP. The *comQXP* operon is constitutively transcribed with no known feedback control or additional regulation. The *comX* gene product is a Pre-peptide that is cleaved and prenylated by ComQ prior to secretion by an unknown mechanism. There are multiple variants (pherotypes) of the *comQXP* locus in different *B. subtilis* strains, but only a single pherotype is encoded by each strain. The receptor encoded by a specific pherotype is not activated by the ComX produced by a non-cognate pherotype. In some cases, the autoinducer of another pherotype inhibits the receptor from responding to its cognate autoinducer. Such cross inhibition is evident between the autoinducer of the RO-H-1 strain (ComX_RO-H-1_) and the receptor of strain 168 (ComP­_168_) [[2-4](#_ENREF_2)] and we use these interactions in the model and in our experiments.

**Rap-Phr quorum-sensing systems**. In *B. subtilis* strain PY79 used in this work, there are seven Rap-Phr systems and three orphan Rap receptors[[5](#_ENREF_5)]. Of these, four complete Rap-Phr systems (and possibly one of the orphan Rap receptors) regulate ComA activity[[6-8](#_ENREF_6)]. The main functions of the Rap systems are to repress ComA, but the mode of regulation differs between Rap proteins. RapC is both a phosphatase of ComA and it also interferes with ComA DNA binding [[9](#_ENREF_9)]. In contrast, RapF only blocks ComA DNA binding [[10](#_ENREF_10)]. Other Rap receptors (not found in strain PY79) do not prevent ComA DNA binding, but prevent transcriptional activation [[11](#_ENREF_11)]. All known Rap receptors diminish or eliminate their interaction with ComA upon binding of their cognate Phr autoinducer. The product of the *phr* gene is the autoinducer that is secreted via the Sec pathway and further processed extracellularly to produce the mature penta- or hexa-peptide autoinducer. This autoinducer is imported back into the cell by the oligopeptide permease (Opp) system, where it interacts with the cytoplasmic Rap receptor. There is little cross-activation between the Phr autoinducer of one Rap-Phr pair and the Rap receptor of another pair, but some orphan Rap receptors have been shown to interact with Phr autoinducers from other systems. The *rap* and *phr* genes are organized in an operon. In many cases, the *phr* gene also has an intrinsic promoter which is regulated by the Spo0A pathway (which is the target of many of the Rap proteins), while the entire operon is sometimes activated by ComA.

## Modeling *B.subtilis* social behavior during swarming

We assume that the quorum-sensing response (i.e, quorum-sensing signals binding to their receptors and the resulting signal transduction cascade that leads to active ComA) is much faster than the growth dynamics of the cells. This assumption allows us to formulate the quorum-sensing response equations and use their steady state solutions to describe the population dynamics.

## Modeling the *B. subtilis* quorum-sensing response network

We consider a simplified ancestral strain that possesses a single ComPX system and a single Rap-Phr system and model its behavior and the behavior of evolved strains that contain an additional Rap-Phr (ExtraRap) or additional ComPX (ExtraCom) system. As explained above, we assume a set level of the autoinducers and derive the steady state relation between the autoinducers and active ComA. Table ST1describes the nomenclature used for the model variables. We make the following simplifying assumptions concerning the model:

1. We assume that the active form of ComP follows Michaelis-Menten enzyme kinetics for regulation of ComA activity.
2. We assume that both the ComPX and Rap-Phr systems are constitutively expressed, and we ignore the feedback of the Rap-Phr systems.
3. We assume that the Rap receptors dephosphorylate ComA via a Michaelis-Menten enzymatic reaction, ignoring the stoichiometric binding effect.
4. We assume that the additional extra system has its specific signal that activates only its own receptor and this system interacts independently with ComA with respect to whether another system is present.
5. In the modified version of the model, we assume that the autoinducer of the ExtraCom system has a lower affinity than the ancestral autoinducer and it can bind to the ComP receptor of the ancestral strain, leading to competitive inhibition.

The reactions describing the intracellular signal transduction model are therefore:

ComPX

1. Binding of ComX and ComP: $X_{i}+P_{i}\leftrightharpoons\left[ P_{i}X_{i} \right]$ ($X_{i}$ is assumed to be in excess, i=1 in the ancestral strain). Also $P_{i}+\left[ P_{i}X_{i} \right]=P^{tot}$

In the modified model, in which we include cross-inhibition, we also assume:

1. Cross inhibition: $P_{1}+X_{2}\leftrightharpoons\left[ P_{1}X_{2} \right]$ (the conservation equation for $P_{1}$ is changed to: $P_{1}+\left[ P_{1}X_{1} \right]+\left[ P_{1}X_{2} \right]=P^{tot}$

Rap-Phr

1. Binding of Rap and Phr: $R_{i}+Ph_{i}\leftrightharpoons\left[ RPh \right]$ (${Ph}_{i}$ is assumed to be in excess)

ComA

1. Phosphorylation of ComA by the ComPX complex: $A\overset{\left[ P_{i}X_{i} \right]}{\overbrace{\to}}A_{a}$
2. Dephosphorylation of ComA by the Rap systems: $A_{a}\overset{R_{i}}{\overbrace{\to}}A$; $A_{a}+A=A^{tot}$

The equations for the above reactions are:

- 1. $\frac{d\left[ P_{i}X_{i} \right]}{dt}=k_{+}^{P}P_{i}X_{i}-k_{-}^{P}\left[ P_{i}X_{i} \right];P_{i}+\left[ P_{i}X_{i} \right]=P^{tot}$
  2. $\frac{dR}{dt}=-k_{+}^{R}R_{i}{Ph}_{i}+k_{-}^{R}\left[ R_{i}{Ph}_{i} \right]; R_{i}+\left[ R_{i}Ph_{i} \right]=R^{tot}$
  3. $\frac{dA_{a}}{dt}=V_{P}\left( \sum\left[ P_{i}X_{i} \right] \right)\times\frac{A}{A+K_{p}}-V_{dP}\left( \sum R_{i} \right)\times\frac{A}{A+K_{dp}};A_{a}+A=A^{tot}$

In the modified model, we also include the cross-inhibition equation

- 1. $\frac{d\left[ P_{1}X_{2} \right]}{dt}=k_{+}^{P}P_{1}X_{2}-k_{-}^{P}\left[ P_{1}X_{2} \right]; P_{1}+\left[ P_{1}X_{1} \right]+\left[ P_{1}X_{2} \right]=P^{tot}$

Without loss of generality, we can normalize the total level of receptors and ComA to be 1. The steady state solution for the above equations can be readily found, for the various models. In the absence of cross-inhibition, both Rap and Com systems have the same functional effect:

- 1. $\left[ P_{i}X_{i} \right]=\frac{X_{i}}{K_{X}+X_{i}}$
  2. $R_{i}=\frac{K_{Ph}}{K_{Ph}+Ph_{i}}$
  3. $V_{P}\sum\frac{X_{i}}{K_{X}+X_{i}}\times\frac{\left( A^{tot}-A_{a} \right)}{\left( A^{tot}-A_{a} \right)+K_{p}}-V_{dP}\sum\frac{K_{Ph}}{K_{Ph}+Ph_{i}}\frac{A_{a}}{A_{a}+K_{dp}}=0$

Further assuming that phosphorylation and de-phosphorylation occur in the linear regime ($A\ll K_{p},A_{a}\ll K_{dp}$), we can find:

- 1. $A_{a}=\frac{\sum\frac{X_{i}}{K_{X}+X_{i}}}{\sum\frac{X_{i}}{K_{X}+X_{i}}+K\sum\frac{K_{Ph}}{K_{Ph}+Ph_{i}}}$

This equation can be further simplified for the regime in which both repression and activation are strong ($X_{i}<K_{X}, Ph_{i}>K_{Ph}$) to be:

- 1. $A_{a}\propto\frac{\sum X_{i}}{\sum\frac{1}{Ph_{i}}}$

If we consider also the cross inhibition of the added ComX on the endogenous system in the ExtraCom strain and in its co-cultured wild-type, we find that the term $\frac{X_{1}}{K_{X}+X_{1}}$ in eq. 1.8 is changed into

- 1. $\frac{\frac{X_{1}}{K_{X}}}{1+\frac{X_{1}}{K_{X}}+\frac{X_{2}}{K_{XI}}}$

in the expression for the quorum-sensing response of both strains.

## Modeling *B. subtilis* community behavior during swarming

The steady-state results of the above quorum-sensing response model are incorporated into a model that describes the growth dynamics of co-cultured strains during swarming. We make the following simplifying assumptions in this model:

1. We use here a highly simplified model of swarming behavior, in which we assume that the production of surfactin allows the population to expand radially into a new volume at a speed $v$, which is proportional to the concentration of surfactin. This behavior exposes new nutrients that allow the community to grow further. Maximal volume is limited to $V_{mx}$.
2. We assume that the community is well mixed throughout its growth volume, preventing the complexity of spatial modeling. Similar simplifying models have been previously used successfully to study social interactions during swarming [[12](#_ENREF_12)].
3. We assume that surfactin production is a non-linear function of ComA activity. We also assume that the *comA* mutant exhibits residual surfactin production. This assumption reflects the growth of the mutant on an effective volume which is considerably larger then the inoculation size, due to inward nutrient diffusion.
4. Cost of quorum-sensing response: We assume that the quorum-sensing response diverts a fraction of nutrient uptake to its production and therefore has a linear influence on growth rate.
5. We assume that all autoinducers are constitutively produced by cells that encode the respective quorum-sensing systems. Autoinducers degrade with an equal time-scale. Autoinducers, surfactant, and nutrients are diluted due to volume expansion of the community.

The equations describing this model are the following:

- 1. Available growth volume: $\frac{dV}{dt}=\frac{2\sqrt{\pi V}vE}{V}, V<V_{mx}$
  2. Usable nutrient, exposed by surfactant and used by cells: $\frac{dN_{s}}{dt}=V_{m}2\sqrt{\pi V}vE-\beta_{N}\frac{N_{s}/V}{1+N_{s}/V}\left( N_{anc}+N_{extra} \right)$
  3. Surfactin: $\frac{dE}{dt}=f_{anc}\left( \frac{X_{1}}{V},\frac{Ph_{1}}{V} \right)N_{anc}$

$+f_{extra}\left( X_{1}/V,Ph_{1}/V,S_{extra}/V \right)N_{extra}-\beta_{E}E$

- 1. Autoinducers: $\frac{dS_{i}}{dt}=\beta_{s}\left( p_{s}\times N_{S}-S_{i} \right)$; This equation holds for $X_{1},Ph_{1}$ and the additional novel signal of the 'Extra' strain. $N_{S}$ is equal to $N_{anc}+N_{extra}$ for the common signals and to $N_{extra}$ for the novel signal.
  2. Ancestral strain population**:**

$\frac{dN_{anc}}{dt}=\frac{N_{s}/V}{1+N_{s}/V}\left( 1-rf_{anc}\left( X_{1}/V,Ph_{1}/V \right) \right)N_{anc}$

- 1. 'Extra' strain population:

$\frac{dN_{extra}}{dt}=\frac{N_{s}/V}{1+N_{s}/V}\left( 1-rf_{extra}\left( X_{1}/V,Ph_{1}/V, S_{extra}/V \right) \right)N_{extra}$

Here, $S_{extra}$ is the autoinducer of the 'Extra' strain – either an additional Phr or ComX. $f_{anc}$, $f_{extra}$ are the quorum-sensing response functions of the ancestral and 'Extra' strain derived from eqs. 1.8-1.10, respectively. We generally use the function $f=f\left( A_{a} \right)=p_{mn}+\left( p_{mx}-p_{mn} \right)\frac{A_{a}^{m}}{K_{a}^{m}+A_{a}^{m}}$. The variables and their values are described in Table ST1. For the behavior of a *comA* mutant, we simply assume $A_{a}=0$. The surfactant is therefore secreted only at its basal level. We assume that the initial population level is $N_{anc}\left( 0 \right)=N_{0}f_{r}, N_{extra}\left( 0 \right)=N_{0}\left( 1-f_{r} \right)$, where $f_{r}$ is the initial frequency of the two strains.

| Variable/Parameter |  | Description | equation |
| --- | --- | --- | --- |
| $X_{1}\left( 0 \right),X_{2}\left( 0 \right),$  $Ph_{1}(0),Ph_{2}(0)$ | 0 | Initial autoinducer level | 1.14 |
| $E\left( 0 \right)$ | 0 | Initial surfactin level | 1.13 |
| $N_{s}(0)$ | 1 | Initial nutrient level | 1.12 |
| $V\left( 0 \right)$ | 1 | Initial (inoculation) volume | 1.11 |
| $N_{0}$ | 0.01 | Initial total inoculum cell number | 1.15,1.16 |
| $v$ | 0.4 | Expansion speed | 1.11,1.12 |
| $V_{mx}$ | 8000 | Maximum volume | 1.11 |
| $V_{m}$ | 1 | Nutrient per volume (equal to $N_{s}(0)/V\left( 0 \right)$ | 1.12 |
| $\beta_{n}$ | 1 | Maximum growth rate | 1.12 |
| $\beta_{E}=\beta_{S}$ | 1 | Degradation time of surfactant and autoinducer | 1.13,1.14 |
| $p_{S}$ | 2 | Production rate of autoinducer per cell | 1.14 |
| $r$ | 0.4 | Cost | 1.15,1.16 |
| $p_{mn}$ | 0.05 | Minimal surfactin production | 1.15,1.16 |
| $p_{mx}$ | 1 | Maximal surfactin production | 1.15,1.16 |
| $m$ | 1 | Hill coefficient of surfactant response to ComA | 1.15,1.16 |
| $K_{a}$ | 0.2 | Hill factor | 1.15,1.16 |
| $K_{ph}$ | 0.01 | Affinity of Phr autoinducer for its receptor | 1.8 |
| $K_{x1}$ | 1 | Affinity of ancestral strain ComX for its receptor | 1.8 |
| $K_{x2}$ | 0.1 | Affinity of novel ComX for its cognate receptor | 1.8 |
| $K_{ix}$ | 0.02 | Affinity of novel ComX fore the ancestral receptor | 1.10 |
| $K$ | 100 | Maximal repression by Rap receptors | 1.8 |
| $T_{mx}$ | 800 |  | 1.11-1.16 |

Figure 2 of the main manuscript shows the results of solving the above equations with the initial conditions listed in Table ST1 over a time $T_{mx}$. S2A Fig shows the final cell density of the different strains. S2B Fig shows the growth curve of the isolated strains as a function of time. S2C Fig shows the change of frequency as a function of time of the derived strains when co-cultured with the wild-type strain. S2D Fig shows the ComA activity level as a function of time for co-cultured wild-type and RapMinus strains.

# Mathematical modeling: *Vibrio harveyi*

*V. harveyi* is a focus of intensive quantitative study of its quorum-sensing network, allowing us to use previous quantitative experimental data and mathematical models in our hypotheses about the social evolution of its quorum-sensing network.

## General information about *V.harveyi* quorum sensing

*V. harveyi* has three quorum-sensing systems[[13](#_ENREF_13), [14](#_ENREF_14)]; the LuxN, CqsS and LuxPQ membrane-bound receptors which bind the autoinducersAI-1, CAI-1 and AI-2, respectively. The three autoinducers are synthesized by the synthases LuxM, CqsA, and LuxS. In the absence of autoinducer, the receptors act as kinases funneling phosphate to LuxU and then to LuxO. In the presence of autoinducers, the receptors act as phosphatases. Phosphorylated LuxO is an active transcription factor that activates the expression of genes encoding four small RNAs, known as Qrr1-4. These four sRNAs repress the expression of several proteins by destabilizing their mRNAs. Most importantly, the Qrr sRNAs repress production of the transcription factor LuxR. LuxR is considered the master regulator of the quorum-sensing response. LuxR-regulated phenotypes include the production of light, which may serve as a public good in the complex ecology of this species. The above network architecture therefore leads to production of LuxR upon addition of all autoinducers. In this regime, all three receptors are phosphatases, LuxO is dephosphorylated and inactive, the *qrr* sRNA genes are not transcribed, and LuxR is derepressed.

## Quantitative analysis of the *V. harveyi* quorum-sensing response.

Previously, Long et al [[15](#_ENREF_15)] measured the promoter activity of *qrr*4 as a function of autoinducer concentration for three strains. The first strain, Δ*luxS*, Δ*luxM* does not produce the AI-1 and AI-2 autoinducers but responds to both. The two other strains were also deleted for either the *luxPQ* or *luxN* genes encoding receptors. These two strains respond exclusively to one of the autoinducers. The authors found that responses could be well approximated by the expressions:

- 1. For the Δ*luxS,* Δ*luxM,* Δ*luxPQ* strain:

$${Pqrr}_{\Delta2}=f_{1}\left( s_{1} \right)=\alpha_{1}+\frac{g_{1}}{1+\frac{s_{1}}{K_{1}}};\alpha_{1}=0.07;g_{1}=1.53;K_{1}=6.9nM$$

- 1. For the Δ*luxS*, Δ*luxM*, Δ*luxN* strain:

$${Pqrr}_{\Delta1}=f_{2}\left( s_{2} \right)=\alpha_{2}+\frac{g_{2}}{1+\frac{s_{2}}{K_{2}}};\alpha_{2}=0.09;g_{1}=1.49;K_{1}=6.4nM$$

They found that the response of the Δ*luxS*,Δ*luxM* strain to both autoinducers was very well approximated as the sum of the separate responses:

- 1. For the Δ*luxS*,Δ*luxM* strain:

$$Pqrr=f_{12}\left( s_{1},s_{2} \right)=f_{1}\left( s_{1} \right)+f_{2}\left( s_{2} \right)$$

Note that while this relation is termed additive by the authors, it is distinct from the additivity of the ComX system. For example, or more generally, of the additivity discussed in section four below. There, we mean that the response is a function of the sum of the autoinducers ($f_{12}\left( s_{1},s_{2} \right)=h\left( s_{1}+s_{2} \right))$, while in the previous *V. harveyi* work it is meant as the sum of the responses of the mutant strains.

The Qrr sRNAs are located at an intermediate tier in the signal transduction cascade that governs the *V. harveyi* quorum-sensing response. In our model, we need to understand the gene expression for those targets that are negatively regulated by the Qrr small RNAs, such as the transcriptional regulator *luxR* or its downstream target the *lux* operon.

Tu et al [[16](#_ENREF_16)] measured LuxR levels (using an RFP-LuxR protein fusion) as a function of autoinducer level when equal amounts of AI-1 and AI-2 were provided (Fig 6A of Tu et al, wild-type line in red). They found that LuxR levels increased with an EC_50_ of 36nM and Hill coefficient of 1.3 from an arbitrary unit value of ~0.25 to ~1.25. If we assume that LuxR levels depend on Qrr levels through the general relation:

- 1. $LuxR\left( Pqrr \right)=\alpha_{R}+g_{R}\frac{1}{1+\left( \frac{Pqrr}{K_{Qrr}} \right)^{n}}=\alpha_{R}+g_{R}\frac{1}{1+\left( \frac{f_{1}\left( s_{1} \right)+f_{2}\left( s_{2} \right)}{K_{Qrr}} \right)^{n}}$

We therefore need to identify the parameters in eq. 2.4 that will best fit the values found by Tu et. al. We find that the two models fit well for a Hill coefficient $n$ in the range of 1.5-2 and an affinity coefficient $K_{Qrr}\sim0.6$. For example, we take the values $\alpha_{R}=0.15,g_{R}=1.25,K_{Qrr}=0.63,n=1.5$. S7 Fig shows the resulting response functions expected from this model for the three strains discussed above. As discussed in [[17](#_ENREF_17)], the response function is affected by additional complications, which we have ignored here including the negative feedback in the system etc. Note the similarity between the expected LuxR response function to the two autoinducers and the measured response (Fig 4B of [[17](#_ENREF_17)]). It is worth noting that the LuxR response curve is not synergistic (per our definition in section 4 below), but some of its regulated responses, such as bioluminescence, are highly synergistic [[18](#_ENREF_18)]. This finding implies that the cost of the quorum-sensing response ($f_{12}$ in eq. 3.2) may be synergistic.

Irrespective of the parameters, the monotonous decreasing dependence of LuxR on Qrr levels implies that:

- 1. $LuxR\left( s_{1},s_{2} \right)=\alpha_{R}+g_{R}\frac{1}{1+\left( \frac{f_{1}\left( s_{1} \right)+f_{2}\left( s_{2} \right)}{K_{Qrr}} \right)^{n}}<LuxR_{\Delta j}\left( s_{i} \right)=\alpha_{R}+g_{R}\frac{1}{1+\left( \frac{f_{i}\left( s_{i} \right)}{K_{Qrr}} \right)^{n}}$

Where i=1,2 and j=2,1, respectively. This previous analysis therefore suggests that the *V. harveyi* network follows the design criteria which allow its evolution by invasion of the wild-type into mutants that lack any one of the quorum-sensing systems.

# Mathematical modeling: Generalizations

## General constraints on the selection for additional quorum-sensing systems

We consider the interaction between two strains – an ancestral strain with a single quorum-sensing system and an evolved strain that has acquired an additional system. We assume that the quorum-sensing network controls the production of a costly public good. The growth rate of the bacteria therefore positively depends on the total level of public goods and it negatively depends on the personal cost of production of the public goods. The equations governing the growth and autoinducer production of the two strains can therefore be written as:

3.1) $\frac{dn_{anc}}{dt}=B\left( f_{1}\left( s_{1} \right)n_{anc}+f_{12}\left( s_{1},s_{2} \right)n_{ev} \right)C\left( f_{1}\left( s_{1} \right) \right)G(n_{i})n_{anc}$

3.2) $\frac{dn_{ev}}{dt}=B\left( f_{1}\left( s_{1} \right)n_{anc}+f_{12}\left( s_{1},s_{2} \right)n_{ev} \right)C\left( f_{12}\left( s_{1},s_{2} \right) \right)G(n_{i})n_{ev}$

3.3) $\frac{ds_{1}}{dt}=\alpha_{1}\left( s_{1} \right)n_{anc}+\alpha_{12}\left( s_{1},s_{2} \right)n_{ev}-\beta_{1}s_{1}$

3.4) $\frac{ds_{2}}{dt}=\alpha_{12}^{'}\left( s_{1},s_{2} \right)n_{ev}-\beta_{2}s_{2}$

The functions $f_{1}\left( s_{1} \right),f_{12}(s_{1},s_{2})$ represent the production rate of public goods per cell in the ancestral and evolved strain, respectively. The benefit function $B$ is a monotonously increasing function of the total public goods produced. The benefit is usually an indirect function of the public goods through its action on nutrient availability and additional differential equations are required to fully express it. The cost function $C$ is a function of the public goods produced by the specific cell. $G$ is a generic function expressing other interdependencies between the cells (such as interactions due to logistic growth). We allow the production rate of the autoinducers to also depend on the autoinducers themselves (autoinduction loop).

Using the above equations we can find the equation for the relative fitness difference between the evolved and ancestral strain during growth, $F=\ln\left( \frac{n_{ev}}{n_{anc}} \right)$:

3.5) $\frac{dF}{dt}=\frac{n_{anc}}{n_{ev}}\frac{d\left( \frac{n_{ev}}{n_{anc}} \right)}{dt}=B\left( f_{1}\left( s_{1} \right)n_{anc}+f_{12}\left( s_{1},s_{2} \right)n_{ev} \right)G\left( n_{i} \right)\left[ C\left( f_{1}\left( s_{1} \right) \right)-C\left( f_{12}\left( s_{1},s_{2} \right) \right) \right]$

If we assume that $B,G$ are positive, then the the evolved strain would have an immediate fitness advantage under the condition:

3.6) $C\left( f_{1}\left( s_{1} \right) \right)>C\left( f_{12}\left( s_{1},s_{2} \right) \right)$

If this conditionis true (or false) for any concentration of the autoinducers, then the total fitness difference integrated over the entire growth process will also be positive (negative). If the sign of the cost difference changes during growth, then the total fitness will depend on the details of the growth process.

## Analysis of invasion under specific cases of interest

We can use the general criterion of eq. 3.6, to examine several specific cases:

1. **Invasion from rarity of the evolved strain**. In this case, we assume that the frequency of the evolved strain is close to zero, $n_{ev}\sim0\ll n_{anc}$. The concentration of the unique autoinducer can therefore be also approximated to $s_{2}\sim0$. The equations describing the growth of the strains are therefore:

3.7) $\frac{dn_{anc}}{dt}=B\left( f_{1}\left( s_{1} \right)n_{anc} \right)C\left( f_{1}\left( s_{1} \right) \right)G(n_{i})n_{anc}$

3.8) $\frac{ds_{1}}{dt}=\alpha_{1}\left( s_{1} \right)n_{anc}-\beta_{1}s_{1}$

3.9) $\frac{dF}{dt}=B\left( f_{1}\left( s_{1} \right)n_{anc} \right)G\left( n_{i} \right)\left[ C\left( f_{1}\left( s_{1} \right) \right)-C\left( f_{12}\left( s_{1},0 \right) \right) \right]$

In this case, the ancestral strain growth dynamics will be equal to its clonal growth dynamics. The frequency of the invading strain will increase if the following invasion condition is maintained:

3.10) $C\left( f_{1}\left( s_{1} \right) \right)>C\left( f_{12}\left( s_{1},0 \right) \right)$

This inequality is equivalent to the condition we cite in the manuscript: In the absence of the novel autoinducer, the novel receptor represses the quorum-sensing response compared to that of the ancestral strain. It is worth noting that the condition for the opposite case, in which the ancestral strain invades from rarity cannot be further reduced from eq. 3.6 and therefore depends on examining the full dynamical behavior of the quorum-sensing response function.

1. **OR-like, additive, regulation of the quorum-sensing response**. If $f_{1}\left( s_{1} \right)=h(s_{1})$ and $f_{12}\left( s_{1},s_{2} \right)=h(s_{1}+s_{2})$ (which is approximately the case for the addition of two ComPX systems), then $f_{12}\left( s_{1},s_{2} \right)\geq f_{1}\left( s_{1} \right)$ for all autoinducer values, and therefore, following eq. 3.6, the evolved strain will be counter selected at any given condition.
2. **AND-like, harmonic or multiplicative, effect on the quorum-sensing response**. If $f_{1}\left( s_{1} \right)=h(s_{1})$ and $f_{12}\left( s_{1},s_{2} \right)=h\left( \frac{1}{\frac{1}{s_{1}}+\frac{1}{s_{2}}} \right)=h\left( \frac{s_{1}s_{2}}{s_{1}+s_{2}} \right)$ (which is approximately the case for the addition of two Rap-Phr systems in *B. subtilis* or the addition of the LuxMN and LuxSPQ systems in *V. harveyi*), then $f_{12}\left( s_{1},s_{2} \right)\leq f_{1}\left( s_{1} \right)$ for all autoinducer values, and therefore, following eq. 3.6, the evolved strain will be selected over the ancestral strain at any given condition.

## The relation between invasion of the evolved strain and the structure of the resulting signal gate

In this section, we will show that accumulation of quorum-sensing systems in the manner we describe, will lead to an AND-like, synergistic effect of the autoinducers, under several biologically relevant conditions. We qualitatively define the quorum-sensing response to be synergistically dependent on two regulating autoinducers if the response when the two autoinducers are added is significantly larger than the sum of the responses when only one of the autoinducers is added. Mathematically, and using the above notation, this means:

3.11) $f_{12}\left( s_{1},s_{2} \right)\gg f_{12}\left( s_{1},0 \right)+f_{12}\left( 0,s_{2} \right)$

We argue that this condition follows from the condition for invasion and three additional assumptions – symmetry, strong repression, and facultative operation. Symmetry means that a strain with multiple quorum-sensing systems will invade from rarity into an 'ancestral-like' strain lacking any of the systems possessed by the wild-type. Strong repression implies that, in the absence of an autoinducer, each receptor promotes a strong reduction in the quorum-sensing response compared to the ancestral-like strain lacking both the receptor and the autoinducer. Finally, facultative operation implies that, in the presence of a sufficient concentration of an additional autoinducer, the strong repressive effect of the additional receptor is fully relieved.

Symmetry implies that:

3.12) $f_{12}\left( s_{1},0 \right)<f_{1}\left( s_{1} \right) ; f_{12}\left( 0,s_{2} \right)<f_{2}(s_{2})$

Strong repression implies that the inequalities are strong:

3.13) $f_{12}\left( s_{1},0 \right)\ll f_{1}\left( s_{1} \right) ; f_{12}\left( 0,s_{2} \right)\ll f_{2}(s_{2})$

Finally, facultative operation implies that:

3.14) $f_{12}\left( s_{1},s_{2} \right)\sim f_{1}\left( s_{1} \right)\sim f_{2}\left( s_{2} \right)$

From the above equations, we can find the required approximation:

3.15) $f_{12}\left( s_{1},s_{2} \right)\gg f_{12}\left( s_{1},0 \right),f_{12}\left( s_{2},0 \right)\to f_{12}\left( s_{1},s_{2} \right)\gg f_{12}\left( s_{1},0 \right)+f_{12}\left( 0,s_{2} \right)$

Note that the conditions of symmetry, strong repression and facultative operation apply for the Rap-Phr system of *B. subtilis* and to a certain extent for *V. harveyi*.

We stress that invasion of an additional quorum-sensing system can occur without having an AND-like synergistic relation between autoinducers and that the other conditions are also strictly required. For example, if $f_{i}\left( s_{i} \right)=\alpha+s_{i}$ and $f_{12}\left( s_{1},s_{2} \right)=\alpha+\frac{s_{1}s_{2}}{s_{1}+s_{2}}$ than the novel system (either system 1 or system 2) will invade (see above discussion), but the condition for synergy is not met if $s_{i}$ is of the order of $\alpha$ or less:

3.16) $f_{12}\left( s_{1},0 \right)+f_{12}\left( 0,s_{2} \right)=2\alpha\geq\alpha+\frac{s_{1}s_{2}}{s_{1}+s_{2}}$

In this case, the strong repression assumption is violated. The response to the autoinducers is sub-additive, but nonetheless, leads to invasion.

In the opposite direction, we can show that synergy and facultative operation are sufficient to ensure invasion by facultative cheating, as eqs. 3.14 and 3.11 imply that $f_{1}\left( s_{1} \right)>f_{12}\left( s_{1},0 \right)$ – the condition for invasion (and the same for autoinducer 2).

Supplementary references

1. Grossman AD. Genetic Networks Controlling the Initiation of Sporulation and the Development of Genetic Competence in Bacillus Subtilis. Annual Review of Genetics. 1995;29(1):477-508. doi: doi:10.1146/annurev.ge.29.120195.002401.

2. Ansaldi M, Dubnau D. Diversifying Selection at the Bacillus Quorum-Sensing Locus and Determinants of Modification Specificity during Synthesis of the ComX Pheromone. J Bacteriol. 2004;186(1):15-21. doi: 10.1128/jb.186.1.15-21.2004.

3. Ansaldi M, Marolt D, Stebe T, Mandic-Mulec I, Dubnau D. Specific activation of the *Bacillus* quorum-sensing systems by isoprenylated pheromone variants. Molecular Microbiology. 2002;44(6):1561-73.

4. Tortosa P, Logsdon L, Kraigher B, Itoh Y, Mandic-Mulec I, Dubnau D. Specificity and Genetic Polymorphism of the *Bacillus* Competence Quorum-Sensing System. J Bacteriol. 2001;183(2):451-60. doi: 10.1128/jb.183.2.451-460.2001.

5. Pottathil M, Lazazzera BA. The extracellular Phr peptide-Rap phosphatase signaling circuit of Bacillus subtilis. Front Biosci. 2003;8:d32-45. Epub 2002/11/29. PubMed PMID: 12456319.

6. Auchtung JM, Lee CA, Grossman AD. Modulation of the ComA-Dependent Quorum Response in *Bacillus subtilis* by Multiple Rap Proteins and Phr Peptides. J Bacteriol. 2006;188(14):5273-85. doi: 10.1128/jb.00300-06.

7. Hayashi K, Kensuke T, Kobayashi K, Ogasawara N, Ogura M. *Bacillus subtilis* RghR (YvaN) represses rapG and rapH, which encode inhibitors of expression of the srfA operon. Molecular Microbiology. 2006;59(6):1714-29.

8. Bendori SO, Pollak S, Hizi D, Eldar A. The RapP-PhrP Quorum-Sensing System of *Bacillus subtilis* Strain NCIB3610 Affects Biofilm Formation through Multiple Targets, Due to an Atypical Signal-Insensitive Allele of RapP. Journal of bacteriology. 2015;197(3):592-602.

9. Lazazzera BA, Solomon JM, Grossman AD. An exported peptide functions intracellularly to contribute to cell density signaling in B. subtilis. Cell. 1997;89(6):917-25. Epub 1997/06/13. doi: S0092-8674(00)80277-9 [pii]. PubMed PMID: 9200610.

10. Baker MD, Neiditch MB. Structural basis of response regulator inhibition by a bacterial anti-activator protein. PLoS-Biology. 2011;9(12):2624.

11. Boguslawski KM, Hill PA, Griffith KL. Novel mechanisms of controlling the activities of the transcription factors Spo0A and ComA by the plasmid‐encoded quorum sensing regulators Rap60‐Phr60 in Bacillus subtilis. Molecular Microbiology. 2015.

12. Xavier JB, Kim W, Foster KR. A molecular mechanism that stabilizes cooperative secretions in Pseudomonas aeruginosa. Molecular Microbiology. 2011;79(1):166-79. doi: 10.1111/j.1365-2958.2010.07436.x.

13. Ng W-L, Bassler BL. Bacterial Quorum-Sensing Network Architectures. Annual Review of Genetics. 2009;43(1):197-222. doi: doi:10.1146/annurev-genet-102108-134304. PubMed PMID: 19686078.

14. Waters CM, Bassler BL. Quorum Sensing: Cell-to-Cell Communication in Bacteria. Annual Review of Cell and Developmental Biology. 2005;21(1):319-46. doi: doi:10.1146/annurev.cellbio.21.012704.131001.

15. Long T, Tu KC, Wang Y, Mehta P, Ong N, Bassler BL, et al. Quantifying the integration of quorum-sensing signals with single-cell resolution. PLoS biology. 2009;7(3):640.

16. Tu KC, Long T, Svenningsen SL, Wingreen NS, Bassler BL. Negative feedback loops involving small regulatory RNAs precisely control the *Vibrio harveyi* quorum-sensing response. Molecular cell. 2010;37(4):567-79.

17. Teng SW, Schaffer JN, Tu KC, Mehta P, Lu W, Ong N, et al. Active regulation of receptor ratios controls integration of quorum sensing signals in *Vibrio harveyi*. Molecular systems biology. 2011;7(1):491.

18. Mok KC, Wingreen NS, Bassler BL. *Vibrio harveyi* quorum sensing: a coincidence detector for two autoinducers controls gene expression. The EMBO journal. 2003;22(4):870-81.
