# Supplementary material for: Social Evolution Selects for Redundancy in Bacterial Quorum Sensing
Source: PLoS Biol. 2016 Feb 29;14(2):e1002386. doi: 10.1371/journal.pbio.1002386 (PMC4771773; doi:10.1371/journal.pbio.1002386)
Supplement: S1 Table — (DOCX) [file pbio.1002386.s010.docx]

Table S1 – strains and plasmids

| Strains | | |
| --- | --- | --- |
| **strain name** | **genotype^a,b^** | **Source and construction method^c^** |
| **B. subtilis** | | |
| AES101 | *B. subtilis* PY79 wild type | *Bacillus* genetic stock center |
| BD2876 (*B. subtilis* 168) | his leu met *srfA*-*lacZ* Tet; *comQ*::Km | kind gift from D. Dubnau |
| AES1974 | ∆*rapF-phrF*::Cm | LFH PCR 🡪 AES101 |
| AES2010 | ∆*rapF-phrF*::Tet | LFH PCR 🡪 AES101 |
| AES1403 | ∆comA::Cm | LFH PCR 🡪 AES101 |
| AES1650 | *sfp::(sfp*_3610_Sp) *swrA::swrA*_3610_ | LFH PCR 🡪 AES101 |
| AES1954 | ∆*rapC-phrC*:: MLS | LFH PCR 🡪 AES101 |
| AES2135 | ∆*comQXP*::Tet | LFH PCR 🡪 AES101 |
| AES2026 | *amyE*::(Psrf-3xYFP Sp) | LFH PCR 🡪 AES101 |
| AES2663 | *ppsB*:: (P*_trpE_*-mCherryPh) | AEC375🡪AES101 |
| AES2539 | *sacA:*:(P*_srf_*-3xYFP Cm) | AEC1003🡪AES101 |
| AES2021 | ∆*rapF-phrF*:: Tet ∆*rapC-phrC*:: MLS | AES1954🡪AES2010 |
| AES2634 | ∆rapF-phrF::Cm ∆*comQXP*::Tet | AES2135🡪 AES1974 |
| AES2543 | ∆*rapF-phrF*::Tet *sacA*::(P_srf_-3xYFP Cm) | AEC1003🡪AES2010 |
| AES2664 | *ppsB*:: (P_trpE_-mCherryPh) ∆*rapF-phrF*::Tet | AES2010🡪AES2663 |
| AES2054 | ∆*comQXP*::Tet *amyE*::(P*_srf_*-3xYFP Sp) | AEC945🡪AES2135 |
| AES2277 | *ppsB*:: (P*_trpE_*-mCherryPh) *amyE*::(P_srf_-3xYFP Sp) | AEC375🡪AES2026 |
| AES2278 | *sacA*::(*comQXP*_RO-H-1_ Cm) *amyE*::(P_srf_-3xYFP Sp) | AEC1018🡪AES2026 |
| AES2202 | ∆*comQXP*::Tet *sacA*::(*comQXP*_RO-H-1_ Cm) *amyE*::(P*_srf_*-3xYFP Sp) | AEC1018🡪AES2054 |
| AES2030 | *sfp::(sfp*_3610_Sp) *swrA::swrA*_3610_ *lacA*::(P_43_-YFP MLS) | AEC767🡪AES1650 |
| AES2137 | *sfp::(sfp_3610_Sp) swrA::swr*A_3610_ *lacA*::(P_43_-YFP MLS) *ppsB*:: (P*_trpE_*-mCherry Ph) | AEC375🡪AES2030 |
| AES2556 | *sfp::(sfp_3610_ Sp) swrA::swrA*_3610_ *lacA*::(P_43_-YFP MLS) ∆*rapF-phrF*::Tet | AES1974🡪AES2030 |
| AES1669 | *sfp::(sfp_3610_Sp) swrA*::*swrA*_3610_ *lacA*::(P_43_-YFP MLS) ∆*comA*::Cm | AES1403🡪AES2030 |
| AES2249 | ∆*rapF-phrF*:: Tet ∆*rapC-phrC*::MLS *amyE*::(*P_hs_-rapC* Sp) | AEC958🡪AES2021 |
| AES2557 | ∆*rapF-phrF*::Tet *amyE*::(P_hs_-*rapF*Sp) *sacA*::(P_srf_-3xYFP Cm) | AEC954🡪AES2543 |
| AES2672 | ∆*rapF-phrF*::Tet *amyE*::(P_hs_-*rapF*Sp) *sacA::(*P*_srf_-3x*YFPCm*) ∆comQ*::kan | BD2876🡪AES2557 |
| AES2513 | ∆*rapF-phrF*::Tet ∆*rapC-phrC*::MLS *amyE*::(*P_hs_-rapC*Sp) sacA::(*P_srf_*-3xYFP Cm) | AEC1003🡪AES2249 |
| AES2636 | ∆*rapF-phrF*:: Tet ∆*rapC-phrC*::MLS *amyE*::(*P_hs_-rapC*Sp) *zjd89*::(P*_hs_-rapF*Sp Km Cm) *sacA*::(*P_srf_*-3xYFP Cm) | AEC954🡪AES2513 |
| AES2871 | *sfp::(sfp*_3610_Sp) *swrA::swrA*_3610_ *lacA*::(P_43_-YFP MLS) *sacA*::(*comQXP*_RO-H-1_ Cm) | AEC1018🡪AES2030 |
| AES2163 | *sfp::(sfp_3610_Sp) swrA::swr*A_3610_ *lacA*::(P_43_-YFP MLS) *ppsB*:: (P*_trpE_*-mCherryPh) *sacA*::(P_rapP_-*rapP^N236T^-phrP* Cm) | AES1656🡪AES2137 |
| AES2522 | *ppsB*:: (P*_trpE_*-mCherryPh) *sacA*::(*comQXP*_RO-H-1_ Cm) | AEC1018 🡪AES2663 |
|  |  |  |
|  |  |  |
| ***V. harveyi*** | | |
| BB120 | Wild-type |  |
| HLS252 | Δ*luxMN* |  |
| BB151 | *luxA::*Tn*5* Km | pBB1131🡪 BB120 |
| JSV950 | Δ*luxMN; luxA::*Tn*5* Km | pBB1131🡪 HLS252 |
|  |  |  |
| **Plasmids^d^** | | |
| AEC375 | *ppsB*::P*_trpE_*-mCherry | [[1](#_ENREF_1)] |
| AEC540 | ece75 | [[2](#_ENREF_2)] |
| AEC541 | ece76 | [[2](#_ENREF_2)] |
| AEC310 | ece174 | [[3](#_ENREF_3)] |
| AEC548 | ece137 | [[4](#_ENREF_4)] |
| AEC954 | pDR111-P_hs_*RapF* | this study |
| AEC958 | pDR111-P_hs_*RapC* | this study |
| AEC945 | pDL30-P*_srf_*-3xYFP | [[5](#_ENREF_5)] |
| AEC1003 | ece174-P*_srf_*-3xYFP | this study |
| AEC767 | ece137-P_43_-YFP | this study |
| AEC1018 | ece174- *comQXP*_RO-H-1_ | this study |
| pBB1131 | pLAFR2/*luxCDABE*::Tn*5* |  |
|  |  |  |
|  |  |  |

^a^All *B. subtilis* strains are in the PY79 genetic background unless otherwise noted.

^b^Resistance markers; Cm, chloramphenicol; MLS, Macrolides-lincosamides-streptogramin B; Km, Kanamycin; Sp, spectomycin; Tet, Tetracyclin; Ph; Phleomycin.

^c^ Marked are the source of strain or construction method. An arrow indicates transformation, trnasduction or conjugation of DNA from the source on the left to the destination on the right.

^d^ All plasmids are maintained in *E. coli* strain DH12 which carries an ampicillin resistance marker.
