# Supplementary material for: Social Evolution Selects for Redundancy in Bacterial Quorum Sensing
Source: PLoS Biol. 2016 Feb 29;14(2):e1002386. doi: 10.1371/journal.pbio.1002386 (PMC4771773; doi:10.1371/journal.pbio.1002386)
Supplement: S2 Table — (DOCX) [file pbio.1002386.s011.docx]

Table S2 – primers used

| name | sequence^a^ | Restriction  site |
| --- | --- | --- |
| rapF-P1 | atcgaggcggctgatcc |  |
| rapF-P2 | Ccggaggtgtagcatgtctcattcaattttgagggttcgttacaccgc  atgtttgga |  |
| rapF-P3 | gcctattttttgtgaatcgattatgtcttttgcgtccatcggcggttttttcg |  |
| rapF-P4 | tcggaccgcacaatgtgt |  |
| rapC-P1 | agtggcttccggcgatt |  |
| rapC-P2 | ctatgagtcgcttttgtaaatttggaaagttataggccgtcccaaccc |  |
| rapC-P3 | ctcggatcccatttccccctttgatttttatgccgccttcatggaga |  |
| rapC-P4 | cggttttggccgcagag |  |
| comA-P1 | aagttggaccggactggaat |  |
| comA-P2 | ttttctaatgtcactaacctgccaaactgttcgctcggttcag |  |
| comA-P3 | agtaatccgcccgacggtatagcggtccattgaatacagc |  |
| comA-P4 | ggtgagccggtgatgtttac |  |
| comQXP-P1 | taaagaccgtatccacttcatgccg |  |
| comQXP-P2 | tgcccgcagctgtgacaaccccctcccattccattttact |  |
| comQXP-P3 | gtagcgcggtggtcccacggctttagatgggcgcct |  |
| comQXP-P4 | ggttggcgttaatctccaaaccaac |  |
| sfp-P1 | ccgccatcctcaccggactt |  |
| sfp-P2 | tgtcttttgcgcagtcggcttcctcaggatctgcccgcc |  |
| sfp-P3 | tcattcaattttgagggttgccaggtgcatacagggtgcctgcc |  |
| sfp-P4 | gcaggagctggaaaagcgcc |  |
| Psrf-sacA-F | atggggaattcgttgtaagacgctc | EcoRI |
| Psrf-sacA-R | cgcggatccttaaagctttttatacag | BamHI |
| comQXP-ROH1-F | agctggatccacaaaagcattgatcagctcga | BamHI |
| comQXP-ROH1-R | agctgaattcagtcgtttccgttataaaaccattaca | EcoRI |
| hsRapF-F | Gatttaagtcgacttatgaagggagggatttgcaaacgtgacaggt  gtcatatcttcttcttcc | SalI |
| hsRapF-R | taatagctagcttagacttcaatttcatacaaactcactcctcct | NheI |
| hsRapC-F | Gatataagtcgacgatgggagagggtgaaggaatgaagagtggg  gtaattcc | SalI |
| hsRapC-R | tatagctagcttagatttcaatttcatacaaaccttcactcc | NheI |

^a^restriction site sequences are underlined in primers that contain them.
